# Minimization of a ship's magnetic signature under external field conditions using a multi-dipole model

**DOI:** 10.1038/s41598-024-58295-1

**Published:** 2024-04-03

**Authors:** Miroslaw Woloszyn, Jarosław Tarnawski

**Affiliations:** Faculty of Electrical and Control Engineering, Gdansk University of Technology, Gdansk, Poland

**Keywords:** Electrical and electronic engineering, Computer science, Computational methods

## Abstract

The paper addresses the innovative issue of minimizing the ship's magnetic signature under any external field conditions, i.e., for arbitrary values of ambient field modulus and magnetic inclination. Varying values of the external field, depending on the current geographical location, affect only the induced part of ship's magnetization. A practical problem in minimizing the ship signature is separating permanent magnetization from induced magnetization. When the ship position changes, a signature measurement has to be made under new magnetic field conditions to update the currents in the coils. This is impractical or even difficult to do (due to the need for a measuring ground), so there is a need to predict the ship's magnetization value in arbitrary geographical location conditions based on the reference signature determined on the measuring ground. In particular, the model predicting the signatures at a new geographical location must be able to separate the two types of magnetization, as permanent magnetization is independent of external conditions. In this paper, a FEM model of the vessel is first embedded in an external field and permanent magnetization is simulated using DC coils placed inside the model. Then, using the previously developed rules for data acquisition and determination of model parameters, a multi-dipole model is synthesized in which the induced and permanent parts are separated. The multi-dipole model thus developed has been successfully confronted with the initial model in FEM environment. The separation of permanent and induced magnetization allows the latter to be scaled according to new values of the external field. In the paper, the situation of determining a signature at one geographical position and its projection onto two other positions is analyzed. Having determined the signature with a high degree of accuracy anywhere in the world, it is possible to perform classical signature minimization by determining DC currents in coils placed inside the ship's hull. The paper also analyzes the effectiveness of ship's signature minimization and the influence of ship's course on the signature value. The advantage of the method presented in this paper is an integrated approach to the issue of scaling and minimization of ship magnetic signature, which has not been presented in the literature on such a scale before.

## Introduction

A ship built of ferromagnetic steel disturbs locally the Earth's magnetic field. This disturbance is referred to as the ship's magnetic signature^1,2^, and for some reasons, such as, for instance, mine protection of a ship with ferromagnetic hull, there is a need to minimize it. It can be achieved by installing current coils inside the ship hull^3^. For this purpose, the Open Loop DegauSsing (OLDS) system is frequently used^4–8^. In this system, the coil currents depending on the ship course are controlled in the open system control. The three-axial magnetometer mounted on the boom above the ship allows to determine the ship's course and pitch, roll, and yaw movements^4^. In numerical FEM modeling of ship’s magnetic signatures, only the induced magnetization is assumed. The authors of^9^ present a method in which ship’s magnetic signatures are first calculated with only one coil activated in the zero external magnetic field, and next, following the superposition method, after adding the induced magnetization in the Earth magnetic field to the ship’s magnetic signature, the coils currents are calculated using the optimization method. The authors of^10^ have compared the application of genetic and particle swarm optimization algorithms for optimizing the ship’s degaussing coil currents. In turn, the authors of^11^ have used the LARS and MPSO optimization algorithms for ship’s signature minimization, while in^12^, the authors have shown that the coil currents can be calculated based on measuring the ship’s magnetic signature without activation of coils and the ship’s magnetic signatures with only one coil activated. In papers^13,14^, the authors have shown that when one of coil currents breaks down it is possible to recalculate the currents in other coils, thanks to which the silencing of magnetic signature is still kept. Another type of ship’s magnetic silencing method is the Close Loop DegauSsing (CLDS) system. In this system, three-axial magnetometers are mounted inside the ship. In papers^15,16^, the authors have shown that sensors mounted inside an open ferromagnetic ship (without deck) allow to minimize its magnetic signatures. In^17^, the CLDS system was used in a double hull submarine. In this case, three-axis sensors were mounted between the internal and external hull of the submarine model. Based on the magnetic field values measured by the sensors, the coil currents took the values that minimized the magnetic signature of the ship. Generally, CLDS systems are used in amagnetic ships (with plastic or austenitic steel hulls)^18^. For an amagnetic ship, silencing of ship’s magnetic signature is achieved by minimizing the magnetic field of any equipment on board the ship^19^.

**Figure 1:**
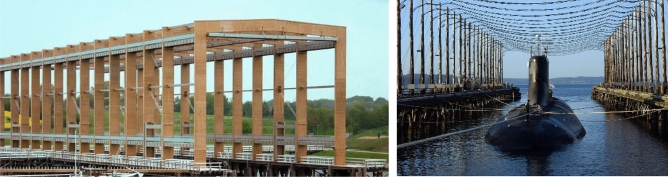
Earth’s magnetic field simulators^20,21^.

The permanent magnetism of a real ship varies in time due to the impact of the hull acting against the water surface, and these changes should be taken into account in the OLDS system. In^20^, the authors present the experimental technique to accurately separate the induced and permanent magnetic field from the total magnetic field generated by a steel ship. This separation was achieved by compensating the Earth's magnetic field by a laboratory magnetic field simulator. In real conditions, using large magnetic field simulators makes it possible to select correct coil currents for different ship positions in the world and to decompose the ship's magnetization (Fig. 1^20,21^).

The authors in this paper do not present an approach that compares it with other existing ones in terms of advantages and disadvantages. The elements of the proposed approach in the paper are based on partial state-of-the-art techniques^18,22–27^. The authors in this paper use the solution developed in their previous works^28^ to scale signatures determined on the measurement range (in the presence of strong permanent magnetization of ship) to any place on the Earth. Reference^19^ concerns signature minimization, but it concerns demagnetization coils wrapped outside an object (e.g. ship engines on trawls), which is a slightly different issue than minimizing the ship's signature using a demagnetization system and coils inside the ship. In the current paper, the authors clarified some details of^28^ and presented a specific example of the use of this scaling, i.e. the possibility of minimizing the signature in any geographical location. In addition to the approach related to the analysis of ship magnetic signature minimization for any geographical location, an analysis related to the ship's course was presented. Innovative rosette charts provide accurate information about the maximum value of the minimized signature. Despite a thorough analysis of the literature, the authors did not encounter such a comprehensive approach. This paper is a development of^28^ and can be used as a decision support system for designing an automatic degaussing system for any ship operating conditions.

To sum up the advantage of the method presented in this paper is an integrated approach to the issue of scaling and minimization of ship magnetic signature, which has not been presented in the literature on such a scale before. The authors' original contribution is the development of the concept of combining the scaling of signatures with significant permanent magnetization with the issue of minimizing the signature predicted in a place other than the one in which the signature was measured. The approach presented in the paper can be useful for the design of automatic ship demagnetization systems in the sense that it allows the selection of the number of coils, their location and the values of coil amperages so that they provide signature minimization at any position in the world and for any heading.

### Numerical ship model

**Figure 2:**
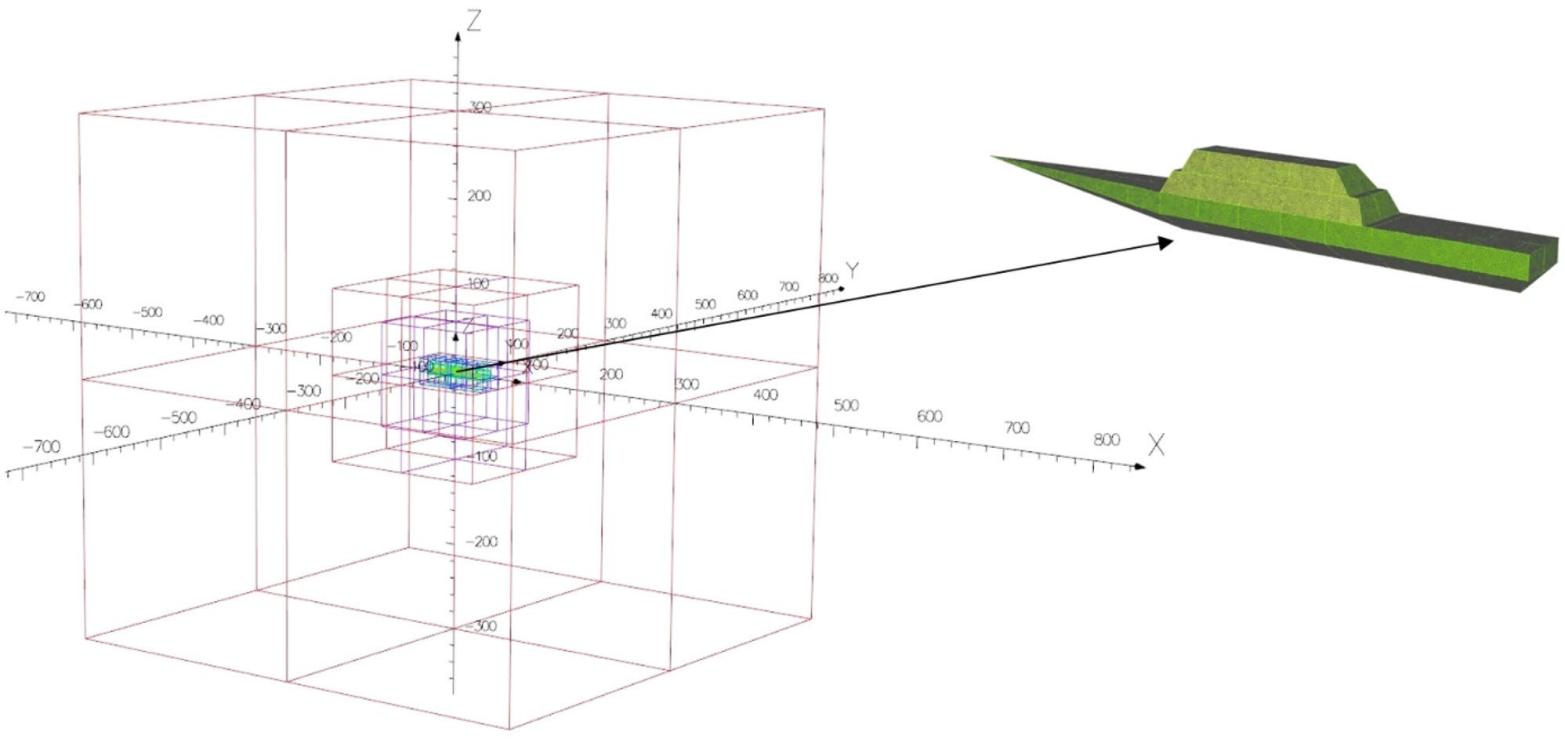
Ship’s model with FEM mesh inside the air-box.

The ship is an object which is difficult to model using the finite element method (FEM), as the thickness of the hull’s steel is thousands times smaller than its overall dimensions (length, width, height). Therefore, FEM needs using a high density mesh to achieve a good shape of finite elements^29–31^. The ferrous ship’s model presented in Fig. 2 was created in the Opera program [opera], which gives the designer the opportunity to model the ship using the thin plate boundary condition method^31,32^. This method is especially useful when the object has much smaller thickness of steel plating than the dimensions. Thanks to it, the mesh sizes can be much greater and the calculation time significantly shorter^32^. The ship model analyzed in this paper has the following dimensions: length—70 m, width—8 m, height—8 m. Moreover, the relative magnetic permeability of *μ*_r_ = 200, the steel thickness of 1 cm, and the isotropy of steel were assumed in the calculations. The relative magnetic permeability of 200 is suitable for ships. The thickness of the hull sheet is usually in the range of 0.5–2 cm. The numerical ship model was placed inside the air-box with sizes 300 m × 300 m × 300 m. The following boundary conditions (1) are introduced for the magnetic scalar potential:

1 $${\mathbf{H}}_{{\text{E}}}=-\nabla \phi $$

where **H**_E_—the magnetic intensity field of Earth, *ϕ*—the magnetic scalar potential. The external magnetic field **H**_E_ with the values of individual components of the vector depending on the value of the angle *φ* (ship's course) was introduced inside the air-box.

The Earth magnetic field is relatively weak (of about 50 μT) and therefore the assumption about the linearity of the magnetic characteristic is justified. A significant problem in ship modeling is permanent magnetization of the ship, which is unknown and, additionally, constantly changing in long time intervals due to mechanical stress of the hull. It also depends on the so-called magnetic history of steel and the vessel building process^1,2^. Permanent magnetization of the ship can be modelled using coils with DC currents placed inside the model^28^. The amper-turns of coils and their positions (Table 1, Fig. 9b) were assumed arbitrary in order to produce a hypothetical permanent magnetization of ship. When an object has only induced magnetization or when permanent magnetization is negligible, scaling the signature and minimizing it is relatively easy. The large proportion of permanent magnetization presented in the paper is due to the fact that the authors want to demonstrate that the demagnetization system proposed in the article can cope even under such difficult conditions. For real ships, the aforementioned demagnetization is carried out to remove permanent magnetization, but the method developed by the authors is suitable for ships containing both types of magnetization.

**Table 1:** Parameters of permanent coils.

	LP1	LP2	LP3	TP1	TP2	TP3	VP1	VP2	VP3
Amper-turns [At]	100	100	100	100	50	120	100	200	100
coil sizes [m]	6 × 1.5	6 × 1.5	6 × 1.5	6 × 1.5	6 × 1.5	6 × 1.5	10 × 4	10 × 4	10 × 4
coil center [m]	(− 2.5,0,0.5)	(− 14.5,0,0.5)	(− 19.5,0,0.5)	(4,0,0.5)	(− 17,0,0.5)	(− 23,0,0.5)	(1,0,0.5)	(− 11,0,0.5)	(− 23,0,0.5)

**Figure 3:**
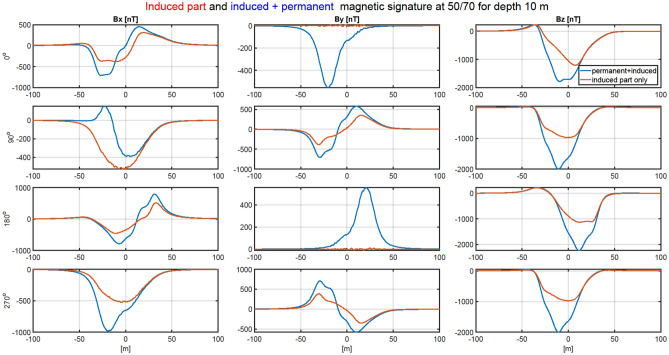
Ship’s signatures (under the keel) for cardinal courses with and without permanent magnetization.

**Figure 4:**
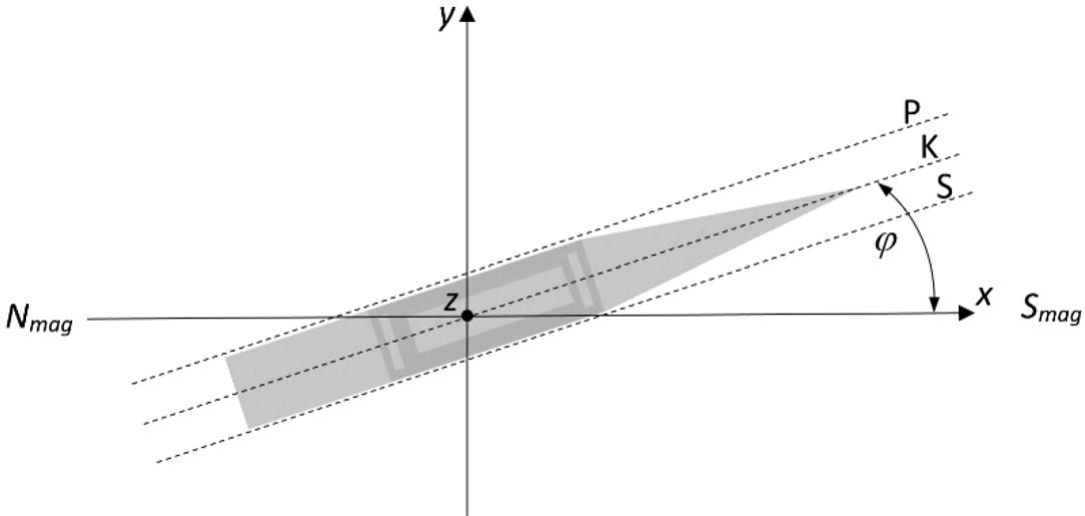
Numerical ship model in Cartesian coordinate system.

For better orientation of the reader, the positions of the characteristic points of the ship are also given. The position of the stern x = − 30 m, the bow x = 40 m, sides ± 4 m. A significant impact of permanent magnetization on the ship magnetic signature was assumed in this paper. Permanent magnetization increases mainly due to the impact of the hull against the water surface. Permanent magnetization can also increase as a result of a moored ship hitting a quay. Permanent magnetization inevitably increases and therefore periodic demagnetization (deperming) is necessary. When an object has only induced magnetization or when permanent magnetization is negligible, scaling the signature and minimizing it is relatively easy. The large proportion of permanent magnetization presented in the article was due to the fact that the authors wanted to demonstrate that the demagnetization system proposed in the article can cope even under such difficult conditions. For real ships, the aforementioned demagnetization is carried out to remove permanent magnetization, but the method developed by the authors is suitable for ships containing both types of magnetization. Figure 3 shows the distribution of three components of the magnetic flux density at depth z = − 10 m under the keel of ship model, for four courses (0°, 90°, 180°, 270°), with and without ship permanent magnetization in the Earth magnetic field BE = 50μT and the magnetic inclination I =  − 70°. The ship signatures with permanent magnetization are about two times greater than those without permanent magnetization. Such a numerical ship model has representative properties for analyzing magnetic signatures and the effectiveness of their minimization for an arbitrary world ship location and course. Figure 4 shows the Port, Keel, Starboard (PKS) paths along which data are collected to determine the model parameters and how the ship's course fi is determined.

When the shape of the ship is symmetrical, e.g. an ellipsoidal ship without permanent magnetization, the signatures actually have a symmetrical shape. However, our model shown in Fig. 2 is not perfectly symmetrical and has permanent magnetization located in various places along the ship, so the shapes of its signature are not symmetrical.

### The separation of ship’s permanent magnetization

**Figure 5:**
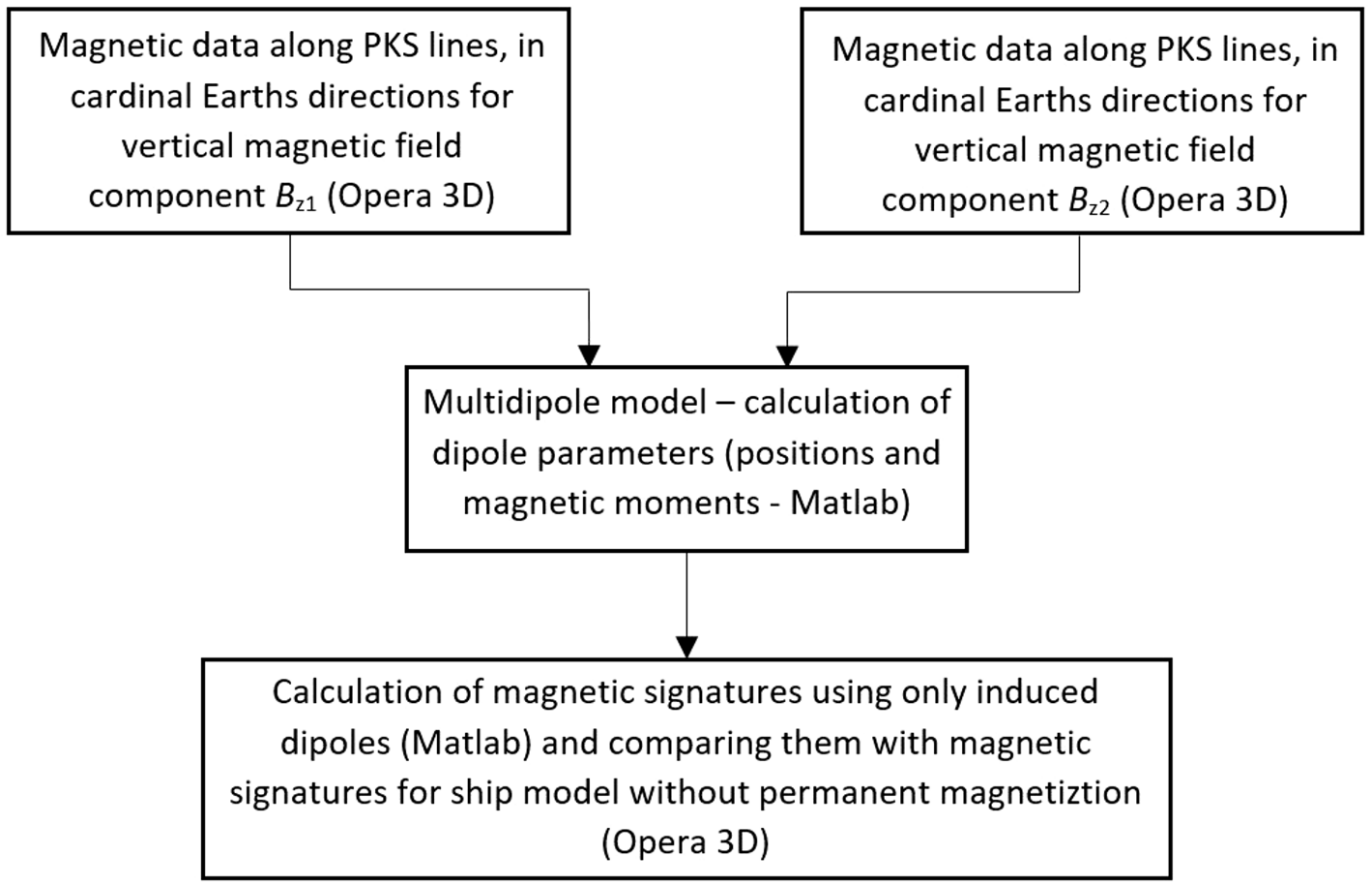
Calculating multi-dipole model parameters and comparing ship’s signatures without permanent magnetization (Opera) with those of the multi-dipole model including only induced dipoles (Matlab).

**Figure 6:**
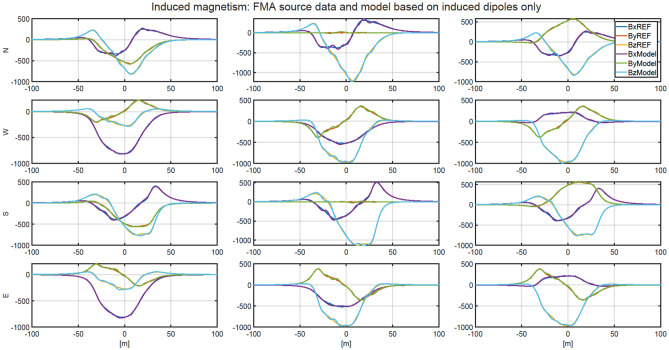
The validation of magnetic signatures of the ship multi-dipole model including only induced dipoles (Matlab) with ships signatures without permanent magnetization (Opera).

The multi-dipole model of the ship allows to separate its signatures related with permanent and induced magnetization^18,22–27,33^. This separation can be obtained using an optimization method which compares the ship signatures measured or calculated using the FEM method with those obtained from the multi-dipole model based on the magnetic data along the PKS lines in cardinal ship courses for two different values of the vertical external magnetic field^28^. The complete magnetic data for the additional value of the vertical external magnetic field is necessary for correct separation of vertical permanent and induced dipoles^28^. The quality of this separation was validated in this paper by comparing the ship signatures obtained from the multi-dipole model (Matlab) for induced dipoles only (as obtained after removing permanent dipoles in the model) with the signatures of the ship model without permanent magnetization calculated in Opera 3D according to the procedure given in Fig. 5. The compared signatures are similar in qualitative and quantitative terms, as shown in Fig. 6. This example proves the correctness of the separation of permanent and induced ship magnetization^28^.

### The minimization of ship’s magnetic signatures

**Figure 7:**
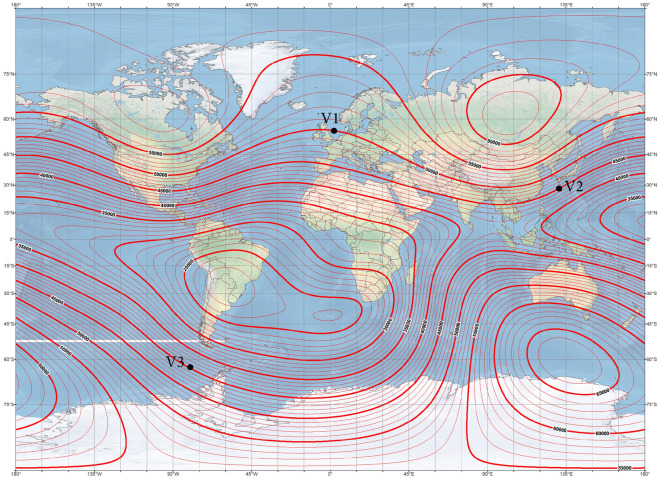
Total magnetic field isoclines at the reference (V1), Japan (V2), and Chile (V3) locations (source: National Centers for Environmental Information^34^).

**Figure 8:**
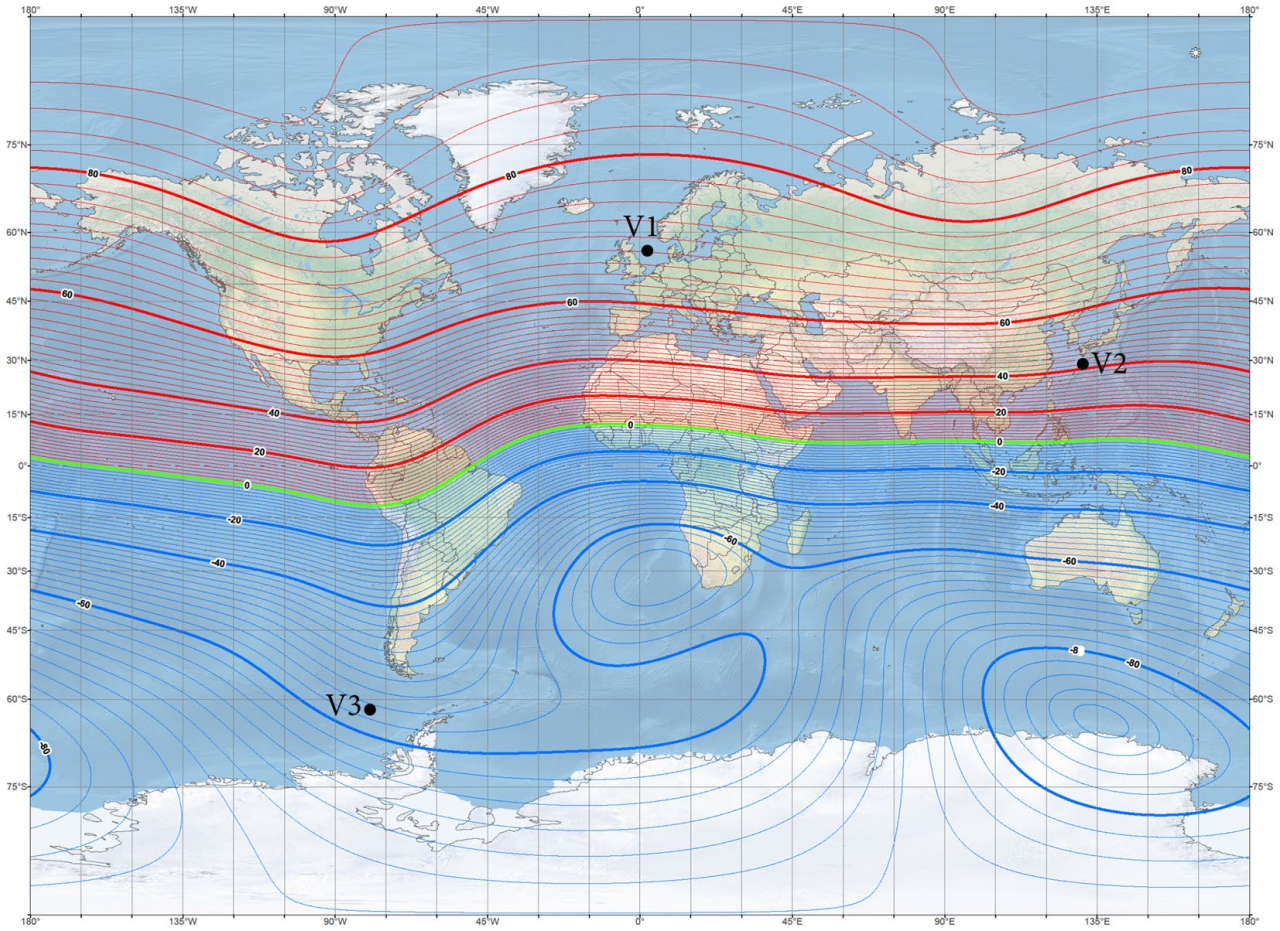
Inclination isoclines at the reference (V1), Japan (V2), and Chile (V3) locations (source: National Centers for Environmental Information^34^).

**Figure 9:**
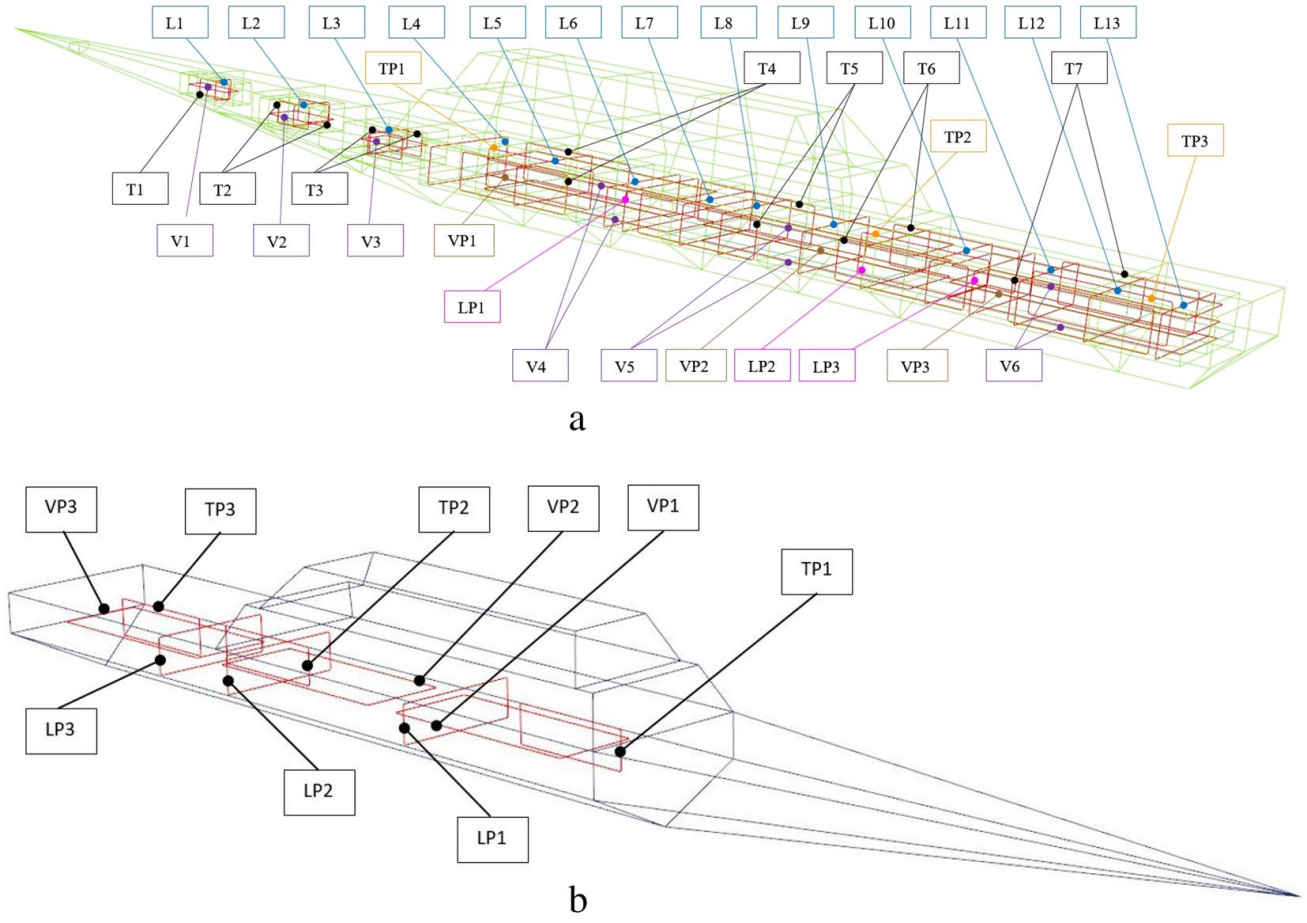
(**a**) Coils inside the ship model. (**b**) Coils related with permanent magnetization inside the ship model.

**Figure 10:**
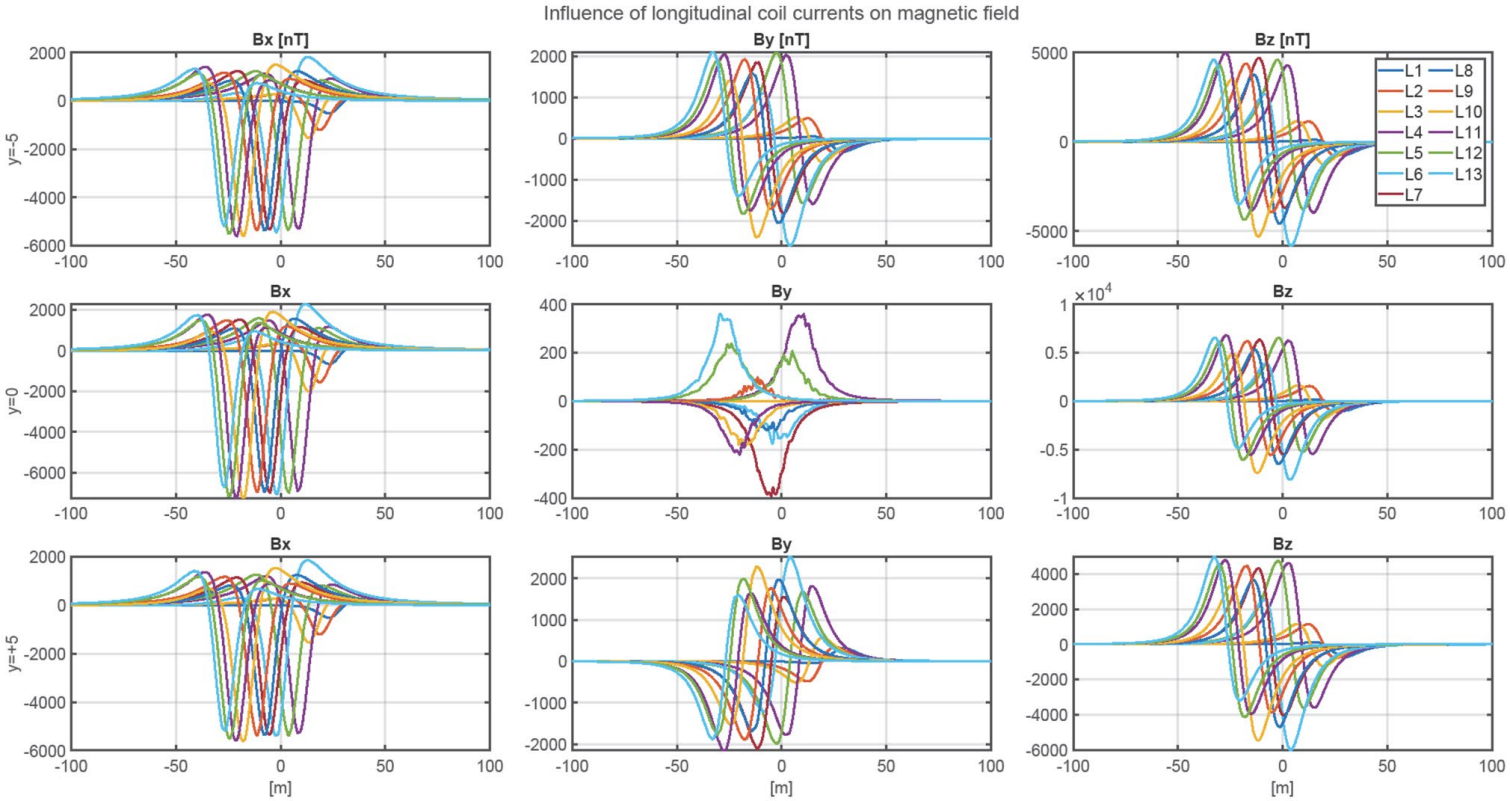
Magnetic flux density of the ship model related with coils L1–L13 (reference current 100 A).

**Figure 11:**
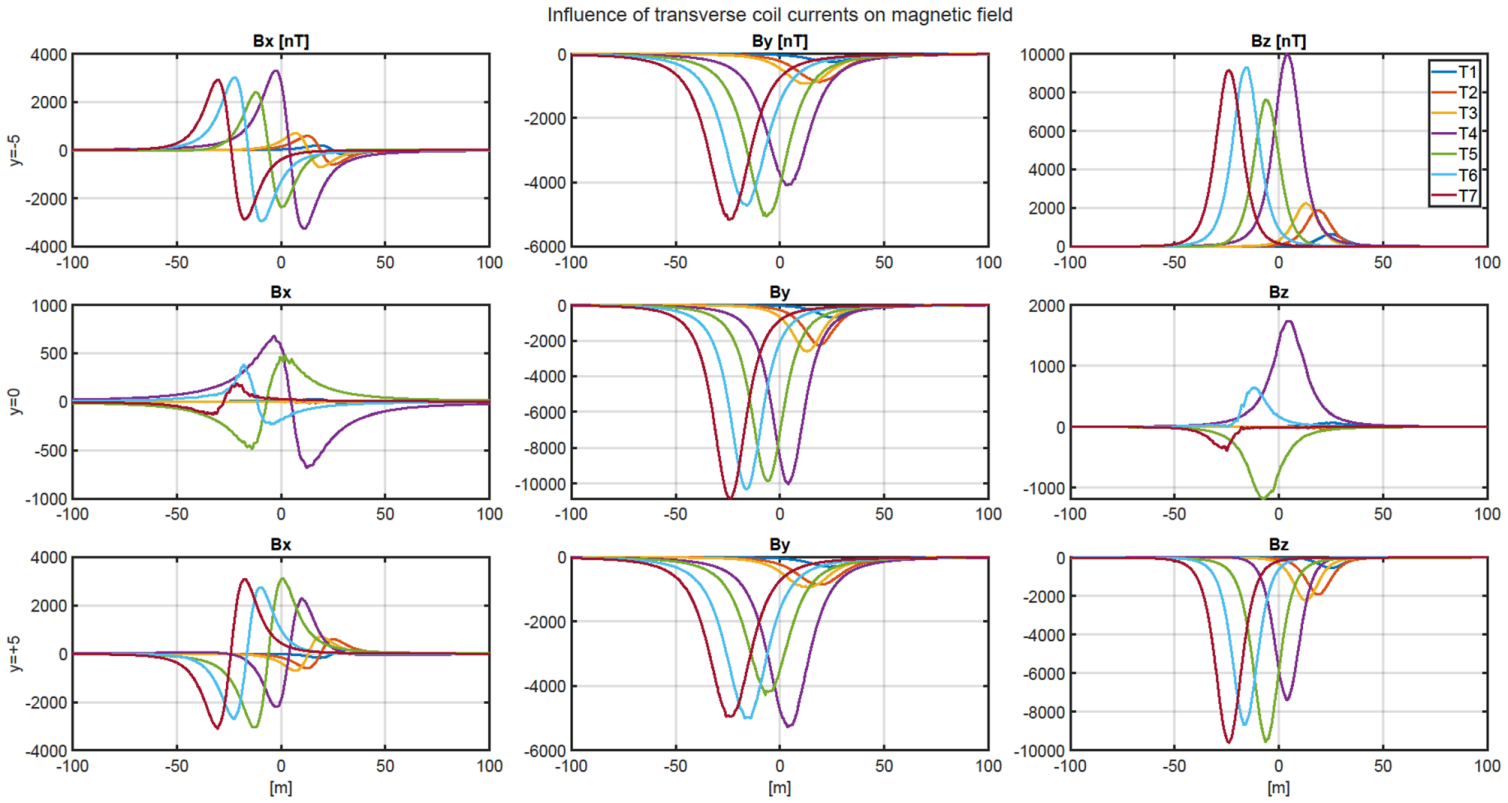
Magnetic flux density of the ship model related with coils T1–T6 (referenced current 100 A).

**Figure 12:**
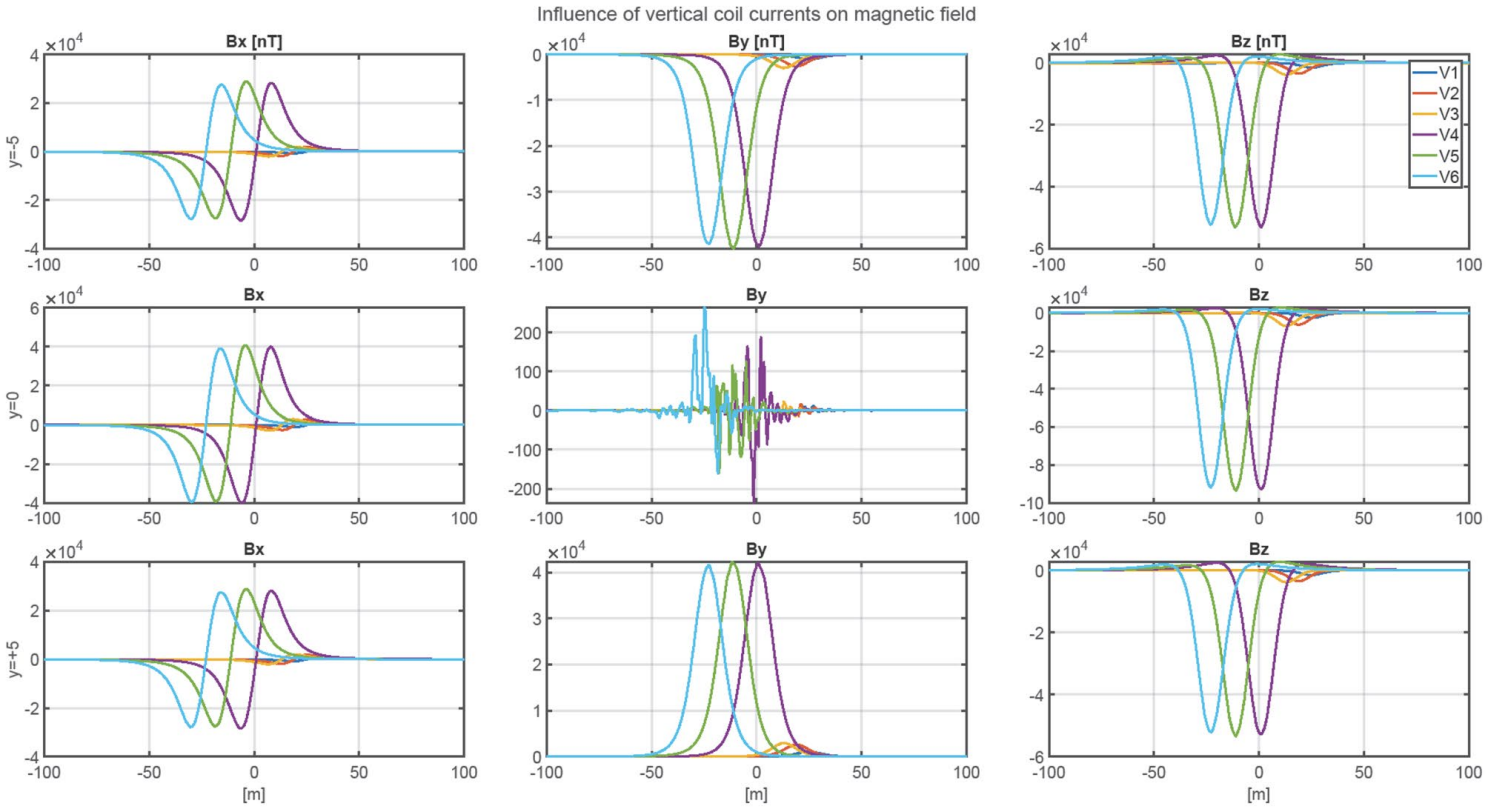
Magnetic flux density of the ship model related with coils V1-V6 (referenced current 100 A).

The ship magnetic signature is minimized by using current coils placed inside the ship^8,12,14,18,19^. The case of ship signature minimization at any world location based only on the induced magnetization was studied in the past by the authors with good results, but such a study is not presented in this paper as a simple benchmark case. Instead, two more complicated cases of minimization of the ship signature model with permanent magnetization are analyzed at two selected geographical locations (hereinafter referred to as Japan V2 and Chile V3—Figs. 7 and 8, respectively). When the parameters of the ship multi-dipole model are determined at one geographical location (reference location V1 in the paper), then it is possible to minimize the ship’s magnetic signatures for an arbitrary ship’s course and location in the world. The authors of^28^ have proved that it is possible to reconstruct the ship’s magnetic signatures at any point in the world when the multi-dipole model of the ship is known. Figure 9 shows the numerical ship model with the demagnetizing coils and nine coils with DC currents (LP1–LP3, TP1–TP3, VP1–VP3) used in the simulations as the equivalent of ship permanent magnetization. The number, sizes, and positions of these coils inside the ship model have not been optimally selected, as it was not the aim of this paper. Determination of number and location of demagnetization coils is an important issue for the signature minimization, but it is a separate and extensive research task. In this article, the authors use the fixed structure described in the article. In total, 26 coils are placed inside the ship model, of which 13 coils L1–L13 generate the longitudinal magnetic field, 6 coils V1–V6 the vertical magnetic field, and 7 coils T1–T7 the transverse magnetic field. The signatures of the ship model which devoid the permanent magnetization related with each coil (the reference current coil was 100 A) are shown in Figs. 10, 11 and 12. The intention of this drawing is to present the local influence of the coils on the magnetic signature of the ship. The distribution of the magnetic field generated by each coil is presented for the area near the coil position. Once the magnetic signatures (26 cases) of the ship model are known, as calculated in the area without an external field related with each coil, then it is possible to determine the optimal coil currents and minimize the ship signature^8,9,13,14^.

The ship’s magnetic signatures for any course and any point in the world can be minimized due to the minimization of the following function (2):

2 $${\text{min}}\sum_{t\in \{P,K,S\}}\sum_{c\in \{x,y,z\}}\sum_{i=(-100:+100)}{\left(\sum_{di=1}^{Nid}{B}_{c,t,i,di}+\sum_{dp=1}^{Npd}{B}_{c,t,i,dp}+\sum_{k=1}^{13}{B}_{c,t,k,i}({I}_{L,k})+\sum_{l=1}^{7}{B}_{c,t,l,i}({I}_{T,l})+\sum_{m=1}^{6}{B}_{c,t,m,i}({I}_{V,m})\right)}^{2}$$

*t*—measurement track along the ship (Portboard, Keel, Starboard) where the signature data are acquired.

*C*—magnetic field density component (*B*_*x*_*, B*_*y*_*, B*_*z*_).

$i$—track coordinate, every meter from − 100 to + 100 m (201 samples per track).

*di*—index of the induced dipole.

$Nid$—number of induced dipoles.

$dp$—index of the permanent dipole.

$Npd$—number of permanent dipoles.

$\sum_{dp=1}^{Npd}{B}_{c,t,i,dp}$—permanent part of ship related magnetic induction generated by coils LP1–LP3, artificial permanent magnetization ${B}_{LPi}, {B}_{TPi}, {B}_{VPi}$

$\sum_{di=1}^{Nid}{B}_{c,t,i,di}$—induced part of ship related magnetic induction originated from Opera FEM software.

${B}_{Li}, {B}_{Ti}, {B}_{Vi}$—magnetic induction related with longitudinal, transverse, and vertical coils, respectively.

${I}_{L,k}, {I}_{T,l}, {I}_{V,m}$—currents in longitude, transverse and vertical coils.

The minimization task is successfully solved by a non-linear least squares method available in any computing package. In Matlab, where the calculations were carried out, it is the *lsqnonlin* function based on the Trust Region Reflective algorithm. In this study, the convergence was obtained after several iterations of the optimization procedure, i.e., very quickly. The influence of initial conditions was practically negligible. The procedure, which was run repeatedly with different initial conditions, did not get bogged down in local minima.

To determine the parameter values of all dipoles, the ship's course was assumed *φ* = 0°. The dipoles locations have to be converted into the Cartesian coordinate system, together with the components of magnetic moments of permanent and induced dipoles regarding the established ship’s course *φ*^23^. The components of the permanent and induced magnetic dipole moments in the Cartesian coordinate system are given by formulas (3) and (4), respectively^24^.

3 $${\mathbf{M}}_{P,i}=\left[\begin{array}{c}{m}_{xP,i}cos\varphi -{m}_{yP,n}sin\varphi \\ {m}_{xP,i}sin\varphi +{m}_{yP,n}cos\varphi \\ {{m}_{zP,i}1}_{z}\end{array}\right]$$

4 $${\mathbf{M}}_{I,j}=\left[\begin{array}{c}{m}_{I1,j}+{m}_{I2,j}{cos}^{2}\varphi \\ {m}_{I2,j}sin\varphi cos\varphi \\ {{m}_{I3,j}1}_{z}\end{array}\right]$$

where *m*_*xP,i*_, *m*_*yP,i*_ and *m*_*zP,i*_ are the *i*-th dipole components of the permanent magnetic moment vector, $\varphi$ is the ship’s course (Fig. 2), and *m*_*I1,j*_, *m*_*I2,j*_, and *m*_*I3,j*_ are the *j*-th dipole aggregated components of the induced magnetic dipole moments.

The components (*m*_*I1,i*_*, m*_*I2,i*_*, m*_*I3,i*_) of the induced magnetic moment vector of the dipole depend proportionally on the Earth’s magnetic field vector^26,28^ (*B*_*Ex*_, 0, *B*_*Ez*_—for course 0°). A linear property of this phenomenon allows to scale the induced magnetic moment (*mʹ*_*I1,i*_*, mʹ*_*I2,i*_*, mʹ*_*I3,i*_) of the dipole^28^ depending on the Earth’s magnetic field values at a new geographical location (*Bʹ*_*Ex*_, 0, *Bʹ*_*Ez*_—for course 0°) (5–7):

5 $${m}_{I1,i}{\prime}={m}_{I1,i}\frac{{B}_{Ex}{\prime}}{{B}_{Ex}}$$

6 $${m}_{I2,i}{\prime}={m}_{I2,i}\frac{{B}_{Ex}{\prime}}{{B}_{Ex}}$$

7 $${m}_{I3,i}{\prime}={m}_{I3,i}\frac{{B}_{Ez}{\prime}}{{B}_{Ez}}$$

where the index *i* represents the *i*-th induced dipole out of the total number *ni* of dipoles.

**Figure 13:**
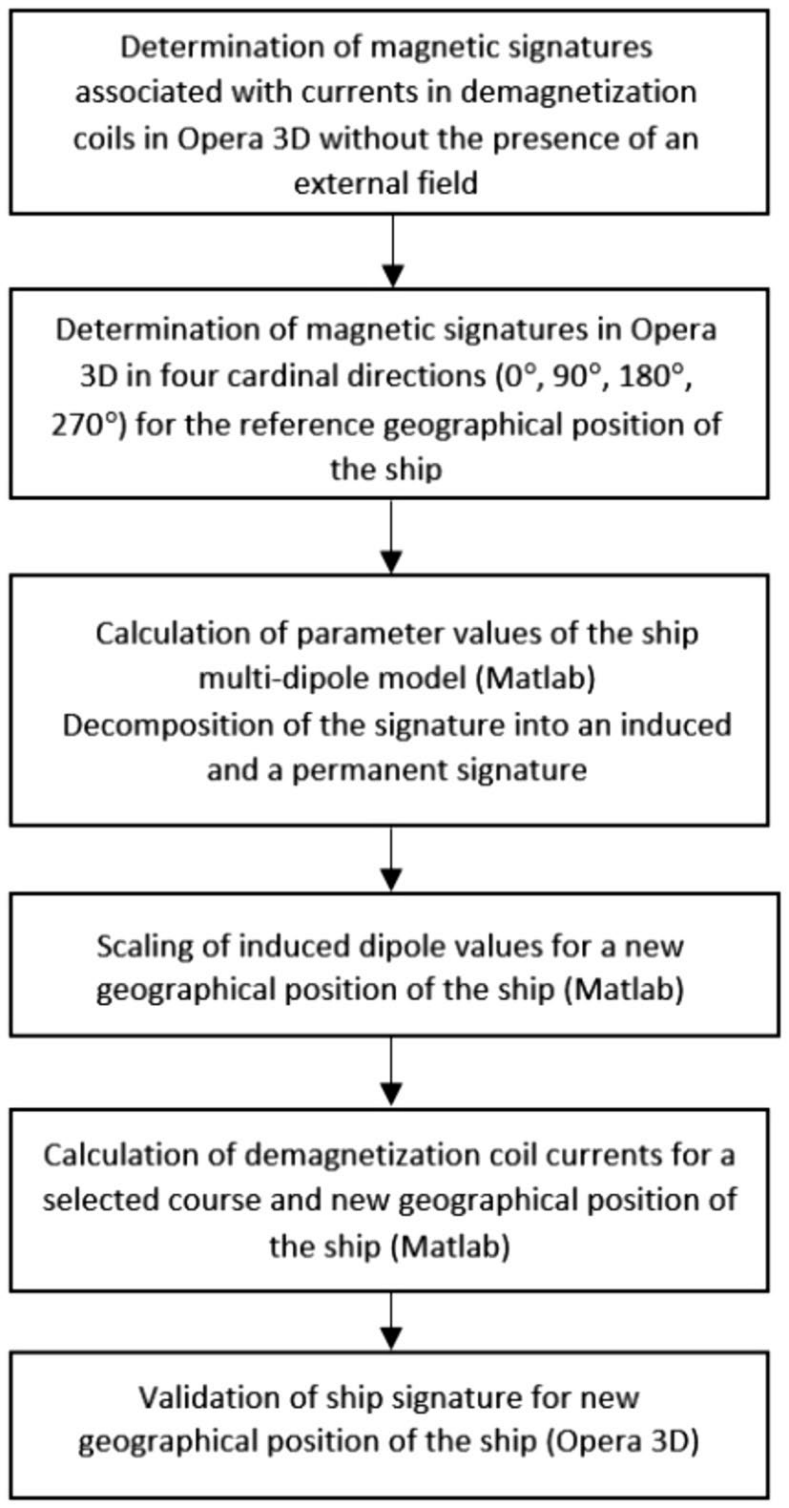
Procedure of minimizing the ship magnetic signature at arbitrary ship location and course.

When the ship’s magnetic signature related with the induced dipoles is recalculated and added to the unchanging signature related with the permanent dipoles, then the resultant signature depends only on the currents in the coils. This means that for a given ship’s course and arbitrary geographical location of the ship, the optimal coil currents minimizing the ship signature can be obtained following the procedure shown in Fig. 13.

### Numerical results of signature minimization

**Figure 14:**
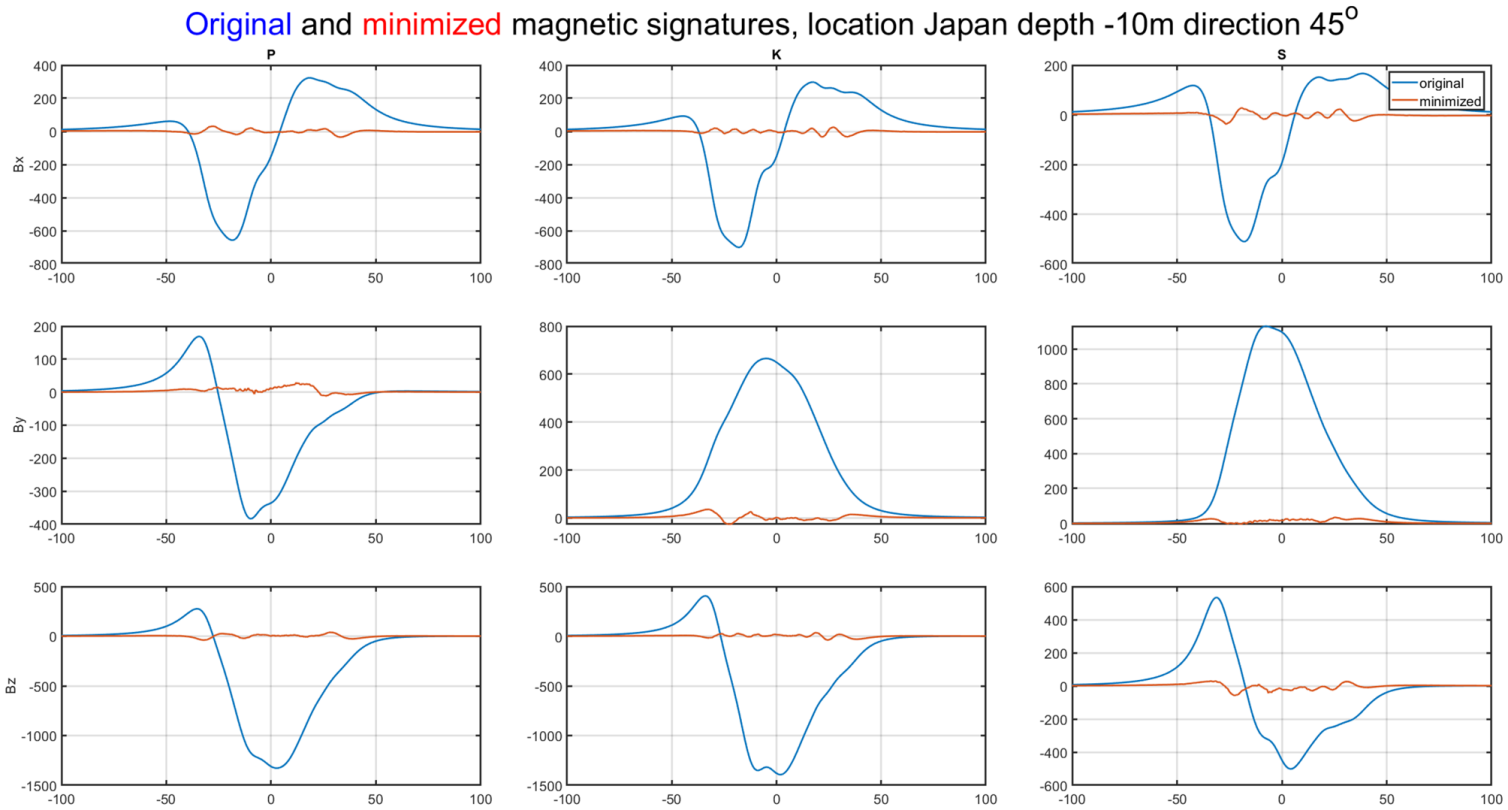
Ship magnetic signatures with and without coil currents at Japan location (V2).

**Figure 15:**
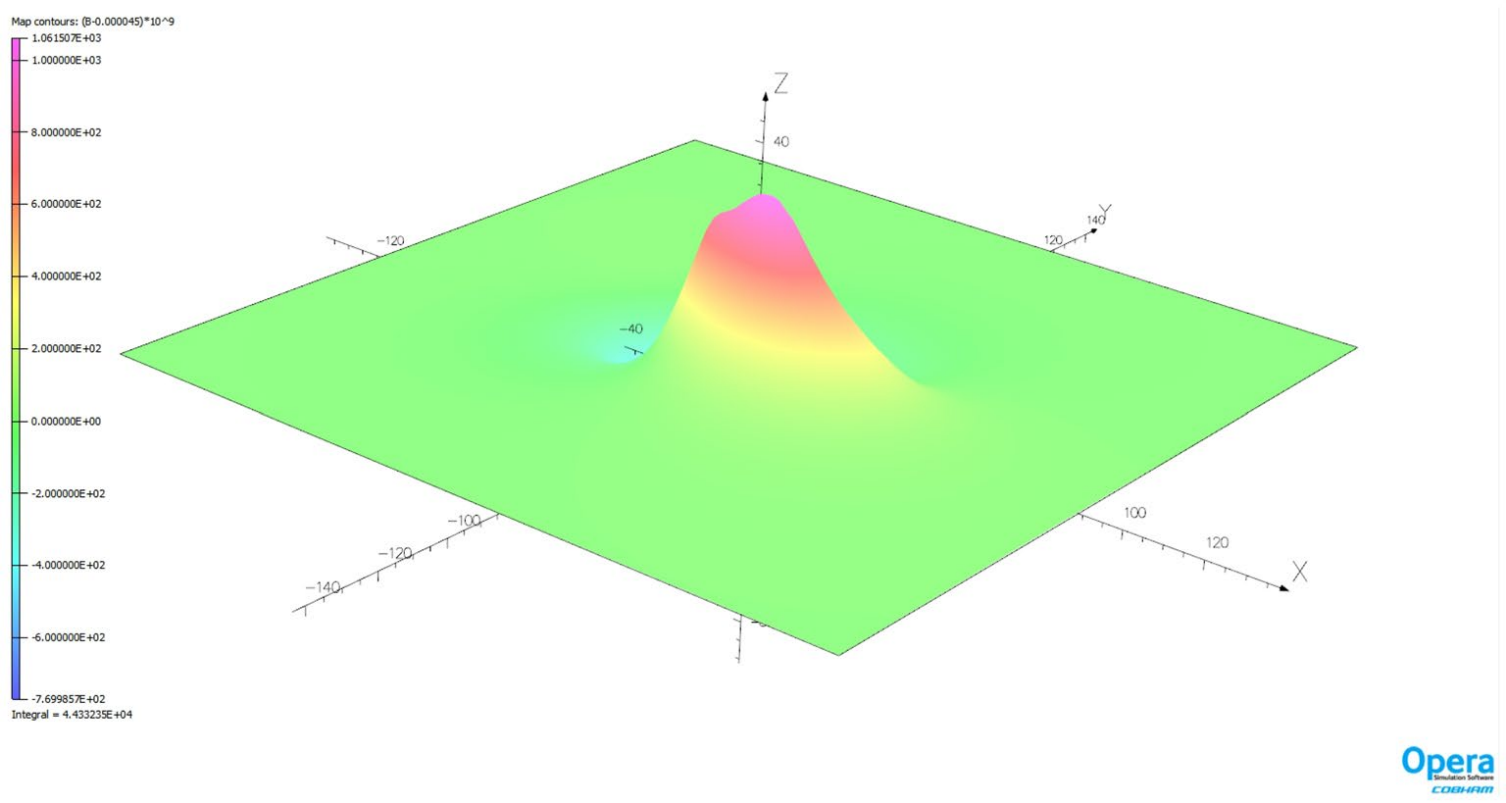
Total magnetic flux density distribution related with the ship model without coil currents (Japan location—V2).

**Figure 16:**
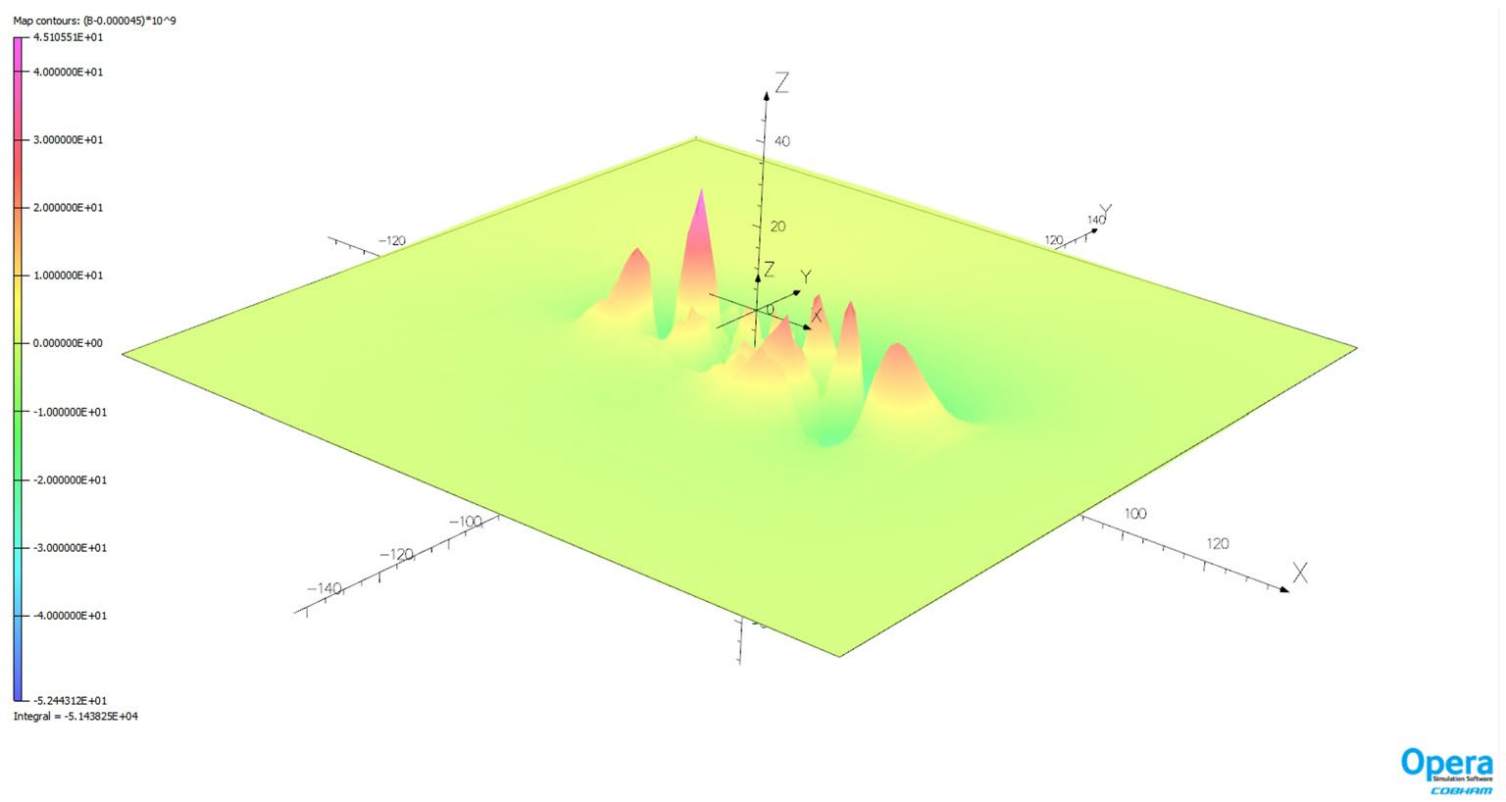
Total magnetic flux density distribution related with the ship model with coil currents (Japan location—V2).

The magnetic signature minimization calculations were performed in the Matlab package using the *lsqnonlin* function based on the *Trust Region Reflective* algorithm. Since a classic hazard in this type of calculation is the influence of initial conditions, 1000 experiments were conducted with random initial conditions. The number of iterations required to complete the calculation and the associated calculation time were also analyzed. The validation of the ship magnetic signature minimization method proposed in the paper was carried out for two selected geographical locations of the ship: V2 and V3. Figure 14 shows the ship magnetic signatures with and without turned on coil currents which were minimized at position V2 near Japan for course 45° and measuring depth *z* = − 10 m. In this case, the minimization of the magnetic signatures was effective. The exact values of the coil currents calculated for this location and ship course are given in Table 2. The mean value and the variance value of the determined currents among 1000 tests are given. The very low variance value indicates that almost the same values were obtained for completely different initial conditions. The total magnetic flux density distributions on the xy plane related with the ship model for the cases with and without coil currents are shown in Figs. 15 and 16 (Opera 3D), respectively. The absolute value of the ship total magnetic field without coil currents is 1061 nT, while for the case with coil currents it is less than 52 nT.

**Table 2:** The values of coils turnampers calculated at Japan (V2) position (means and variances for 1000 different initial conditions).

Coil	L1	L2	L3	L4	L5	L6	L7	L8	L9	L10	L11	L12	L13
Mean	− 3843.9	− 837.0	239.0	− 500.8	− 627.9	− 527.5	− 624.3	51.9	− 821.7	− 470.4	− 563.2	235.9	− 380.7
Variance	1.96e−08	2.35e−08	2.17e−08	9.42e−10	2.42e−10	1.33e−09	6.05e−09	8.56e−09	1.35e−09	3.39e−10	6.21e−10	8.38e−10	2.46e−10

Coil	T1	T2	T3	T4	T5	T6	T7						
Mean	1836.4	222.2	895.2	264.2	413.8	198.7	226.4						
Variance	1.56e−09	5.49e−10	2.24e−10	2.20e−12	1.70e−12	2.67e−13	1.99e−13						

Coil	V1	V2	V3	V4	V5	V6							
Mean	921.2	− 813.6	− 82.3	− 92.7	− 145.4	− 111.8							
Variance	1.74e−09	5.74e−09	4.48e−09	3.86e−12	2.10e−12	2.20e−12

**Figure 17:**
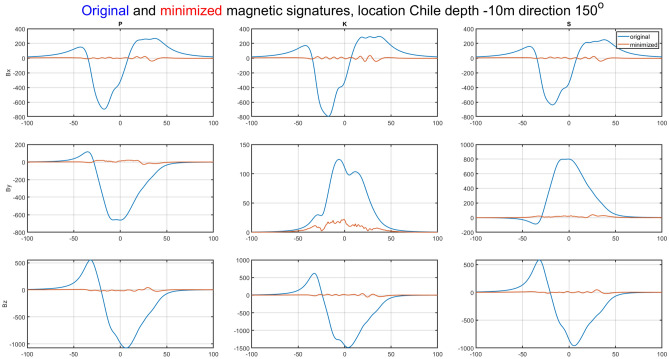
Ship magnetic signatures with and without coil currents at Chile location (V3).

**Figure 18:**
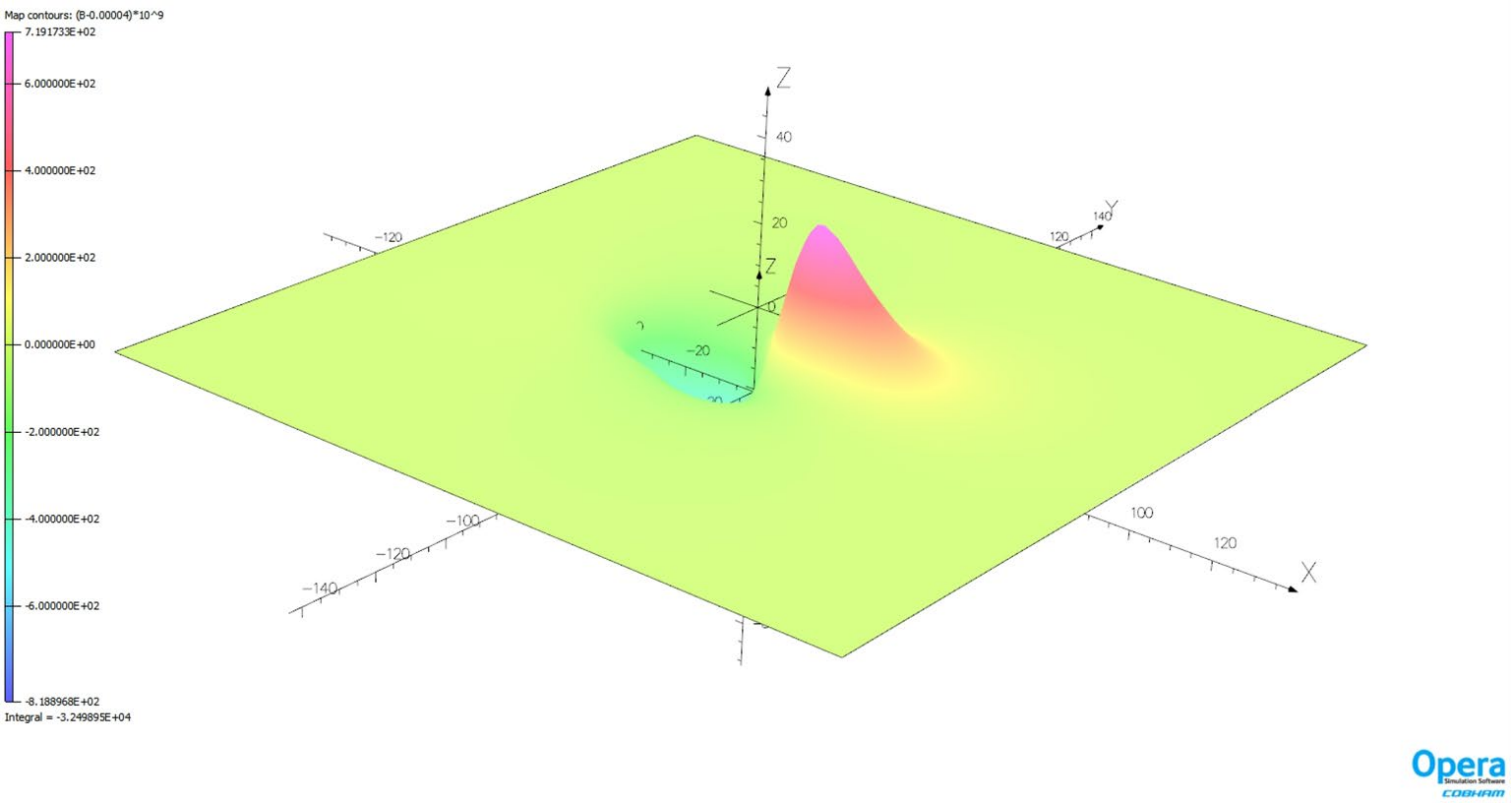
Total ship magnetic flux density distribution related without coil current (Chile location—V3).

**Figure 19:**
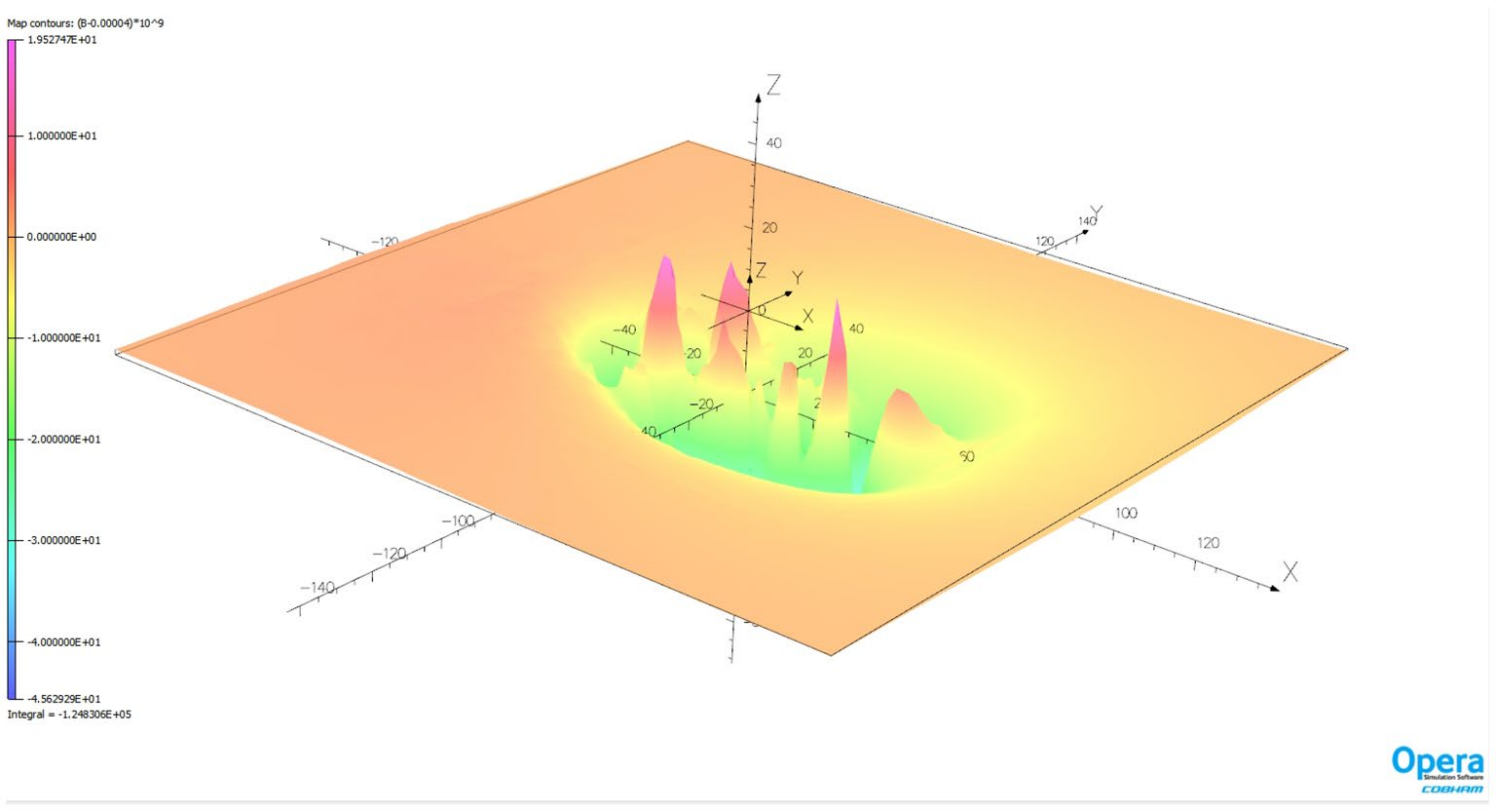
Total ship magnetic flux density distribution related with coil current (Chile location—V3).

The ship magnetic signatures without turned on coil currents and minimized at location near Chile (V3) for course 150° and measuring depth *z* = − 10 m are shown in Fig. 17. Also in this case, the minimization of the magnetic signatures has turned out effective. The exact values of the coil currents calculated for this location and ship course are given in Table 2. The total magnetic flux density distributions on the xy plane related with the ship model for the cases with and without coil currents are shown in Figs. 18 and 19 (Opera 3D), respectively. The absolute value of the ship total magnetic field without coils currents is 818 nT, while for the case with coil currents it is less than 46 nT.

The natural effect of minimizing signatures with DC coils is the formation of the residues seen in Figs. 14, 16, 17 and 19. The distribution of these residues is related to the number of coils and can be minimized by increasing the number of coils. However, this is not practiced because it increases the cost of the demagnetization device, and with minimization we mainly care about the amplitude of the signal, which is effectively reduced.

**Figure 20:**
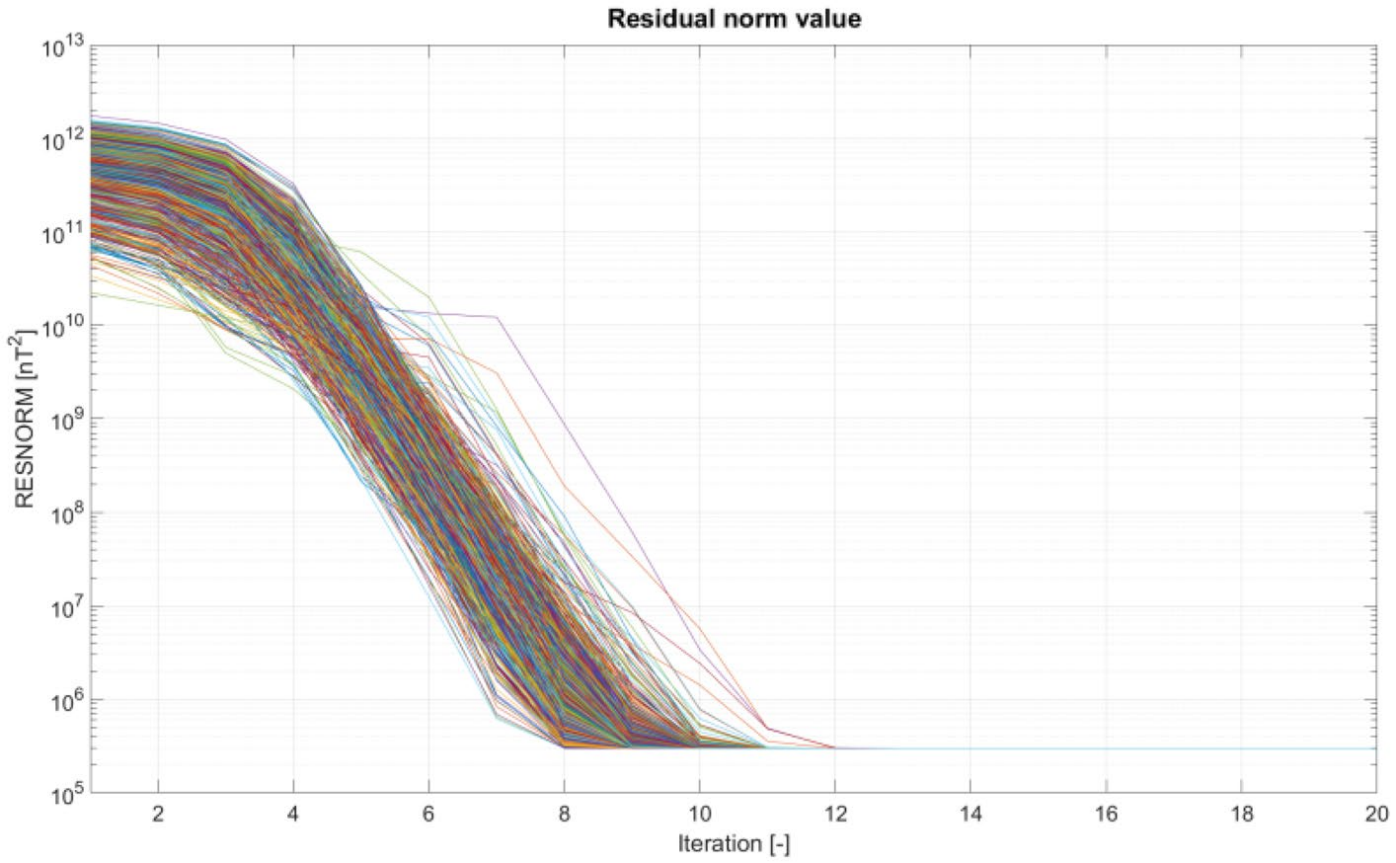
The value of the residue norm indicating the rapid convergence of the algorithm and the independence of the result from the initial conditions.

The effectiveness of magnetic signature minimization at Japan location is about 5.5% (peak to peak of the ship total magnetic field—compare Figs. 15 and 16), while at Chile location it is 4.2% (compare Figs. 18 and 19). It is noteworthy that the sizes and positions of the coils inside the model ship have been assumed arbitrary in the analysis. Optimal selection of sizes and positions of coils inside a ship is an issue worth studying, which, however, goes beyond the scope of this paper. The convergence to the same value of the residue norm was obtained after several iterations of the optimization procedure for different values of initial conditions. The procedure was run repeatedly with different initial conditions and did not get stuck in local minima, as can be observed in Fig. 18. The calculation with 1000 different sets of initial conditions took 344 s. The impact of the initial conditions was negligible as can be seen in Fig. 20 and from the variances given in Tables 2 and 3.

**Table 3:** The values of coils turnampers calculated at Chile position (means and variances for 1000 different initial conditions).

Coil	L1	L2	L3	L4	L5	L6	L7	L8	L9	L10	L11	L12	L13
Mean	2859.6	771.5	− 244.8	373.3	511.5	441.0	− 42.4	532.4	288.7	241.9	180.7	− 16.7	145.5
Variance	3.49e−9	5.02e−9	3.47e−9	3.65e−10	1.02e−10	5.80e−10	2.63e−9	1.85e−9	2.06e−10	5.18e−10	1.17e−9	1.64e−9	1.64e−9

Coil	T1	T2	T3	T4	T5	T6	T7						
Mean	1327.6	60.7	687.2	170.9	245.1	107.4	128.0						
Variance	1.56e−09	5.87e−10	2.34e−10	1.38e−12	1.31e−13	2.68e−13	8.92e−10						

Coil	V1	V2	V3	V4	V5	V6							
Mean	− 715.2	671.5	197.0	0.5	− 41.4	14.8							
Variance	4.31e−10	8.92e−10	8.25e−10	3.33e−12	1.64e−12	4.60e−13							

### Analyzing optimal values of coil currents

**Figure 21:**
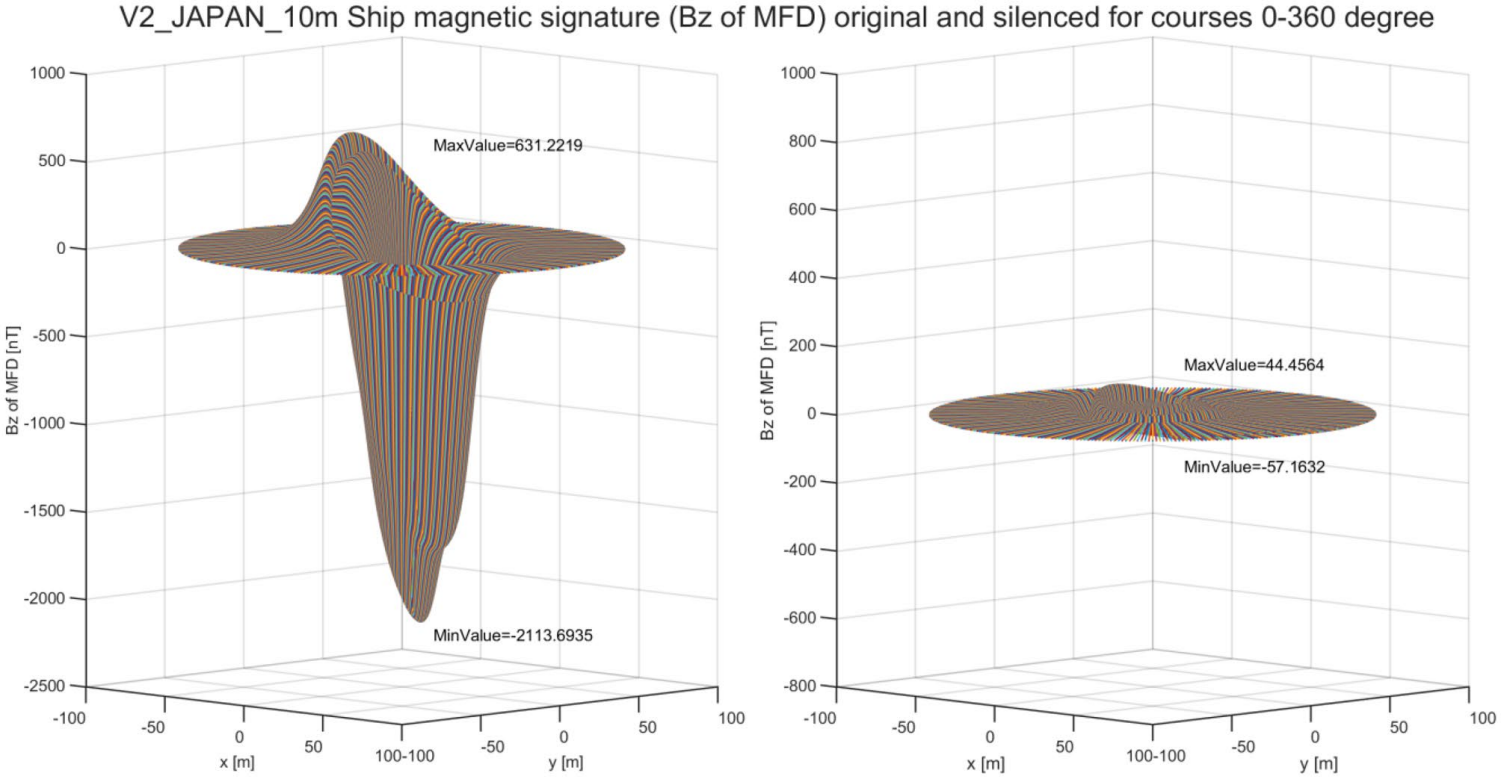
Vertical component of magnetic flux density of the ship model along the line under the keel at Japan location for courses 0°–360°

**Figure 22:**
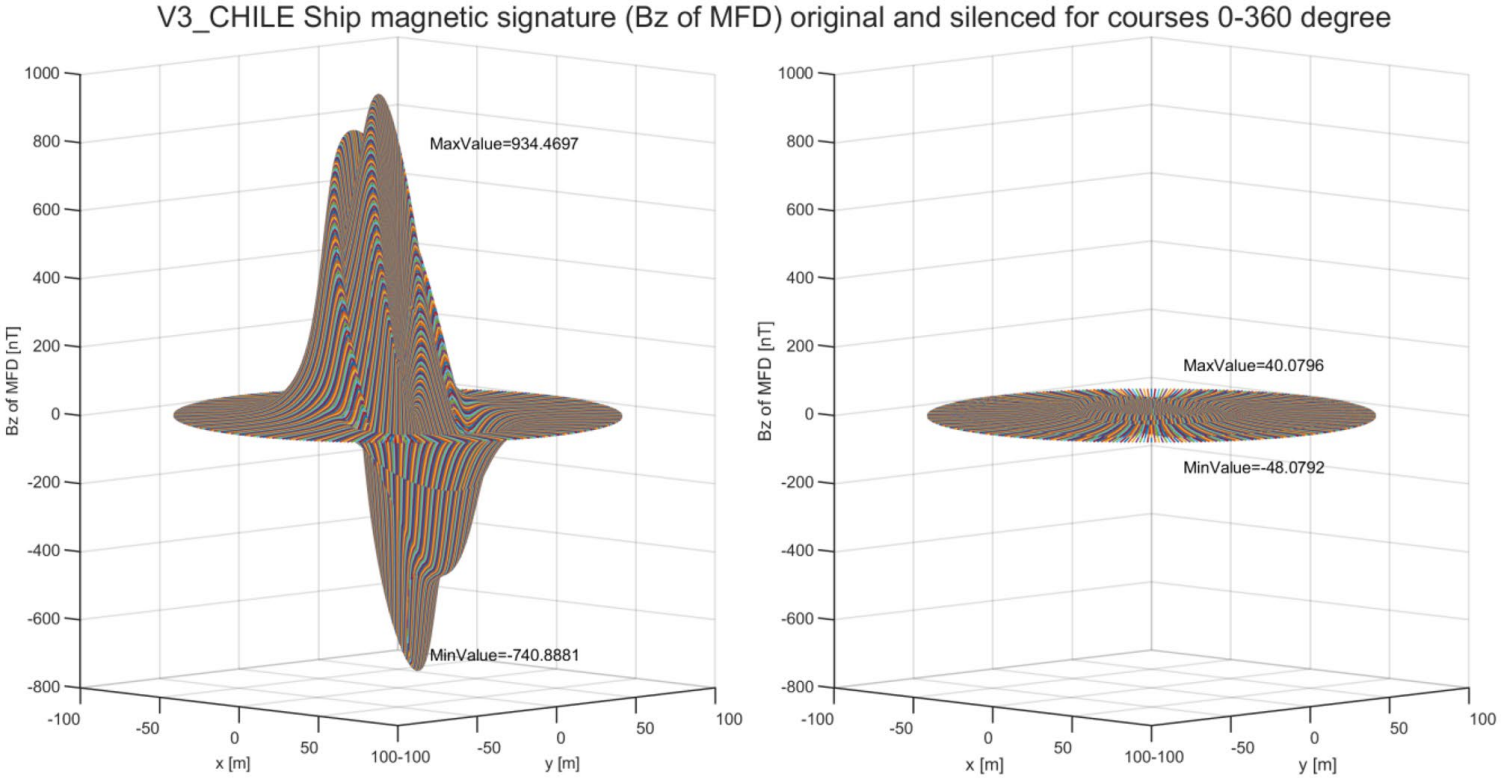
Vertical component of magnetic flux density of the ship model along the line under the keel at Chile location for courses 0°–360°

As said in the previous chapter, the multi-dipole ship model allows to effectively calculate the optimal values of coil currents for signature minimization at any course and geographical location. The next issue analyzed in the paper is the influence of the ship course on the ship magnetic signature. For this purpose, the optimal values of coil currents were calculated at two geographical locations (Japan and Chile) for a complete rotation of the ship model around the vertical axis. The vertical component of the magnetic ship flux density without (left) and with (right) coil currents along the line under the keel for courses 0°–360° at Japan and Chile locations are shown in Figs. 21 and 22, respectively. In both cases, the silencing of the magnetic signatures is effective.

**Figure 23:**
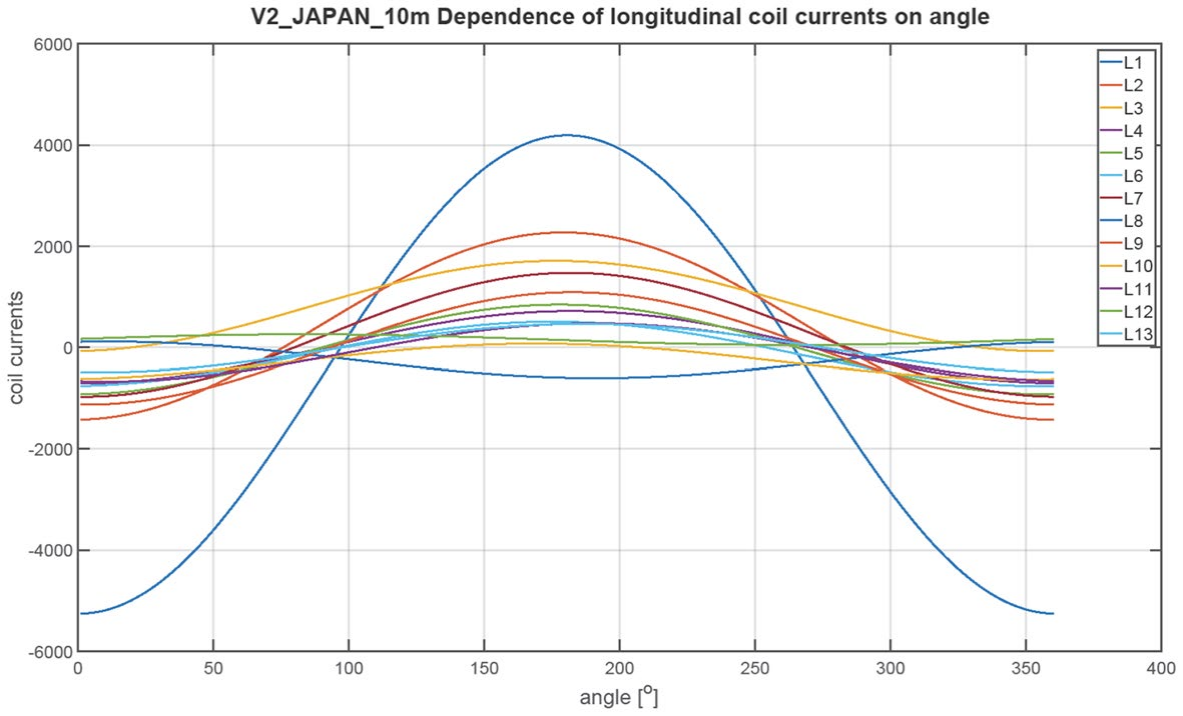
Distribution of currents in L1–L13 coils at Japan location.

**Figure 24:**
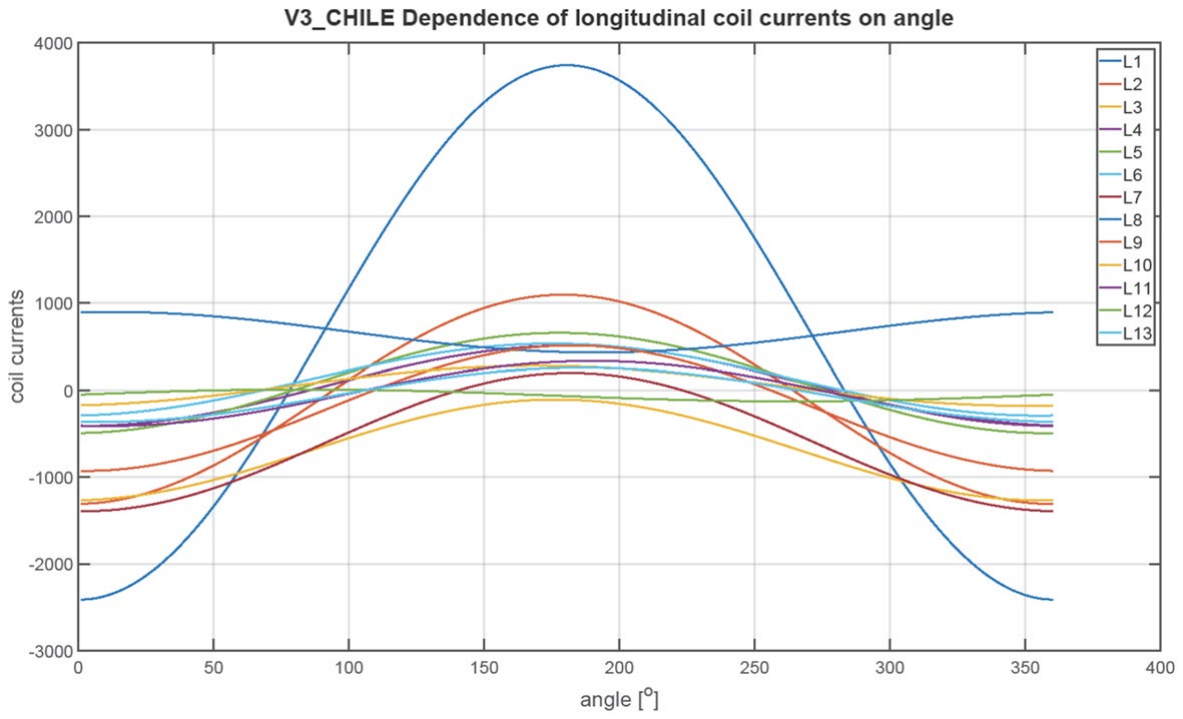
Distribution of currents in L1–L13 coils at Chile location.

**Figure 25:**
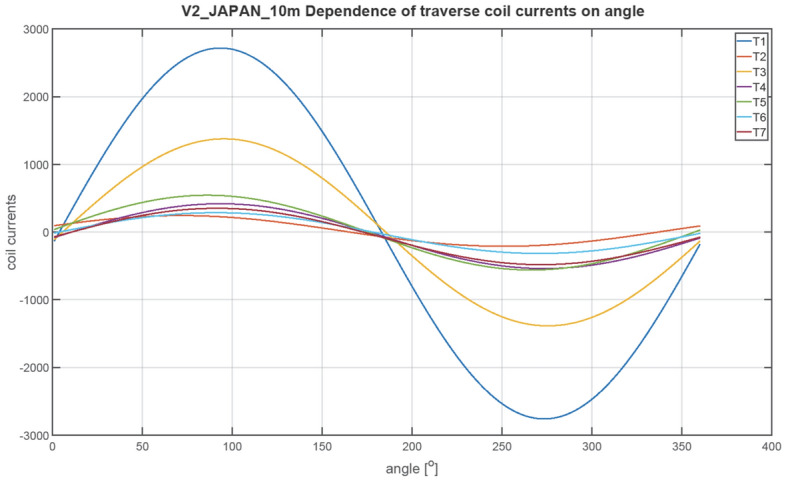
Distribution of currents in T1–T6 coils at Japan location.

**Figure 26:**
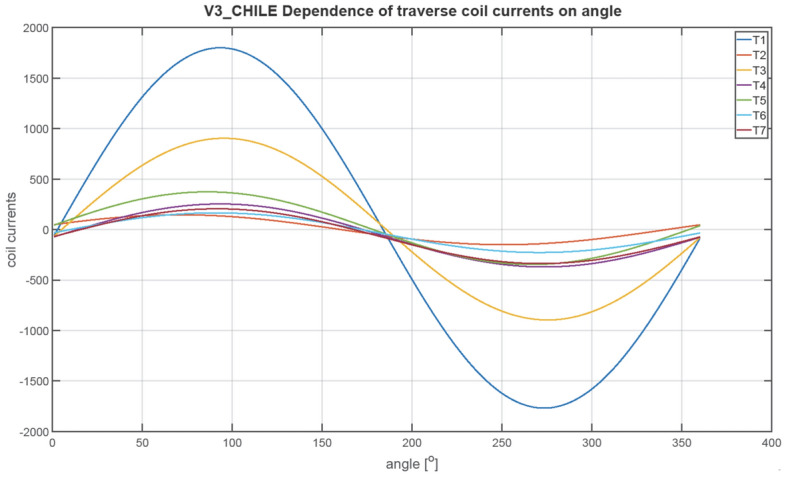
Distribution of currents in T1–T6 coils at Chile location.

**Figure 27:**
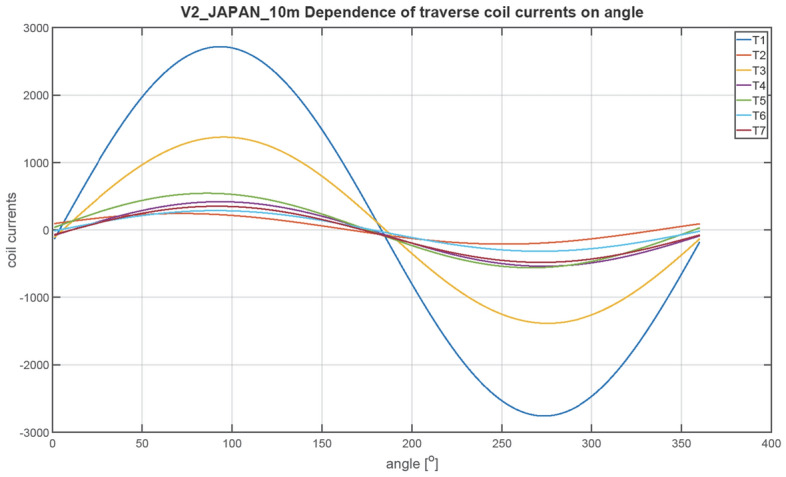
Distribution of currents in V1–V6 coils at Japan location.

**Figure 28:**
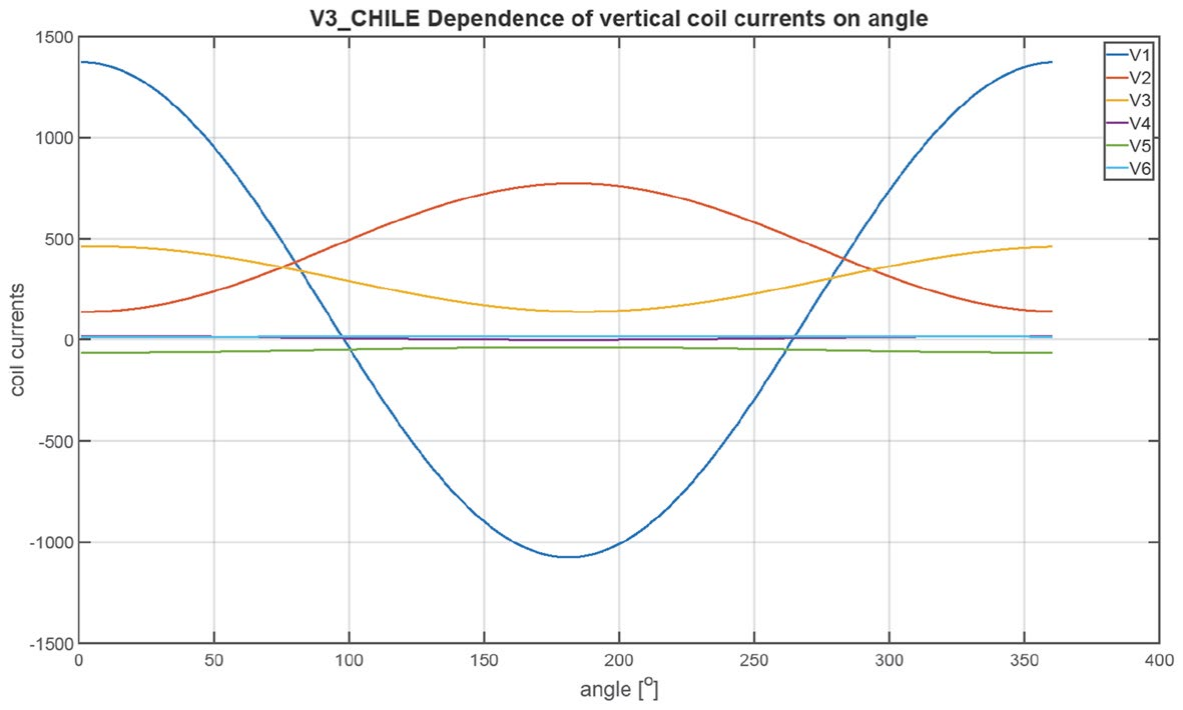
Distribution of currents in V1–V6 coils at Chile location.

The next figures, Figs. 23, 24, 25, 26, 27 and 28, show the distributions of coil currents as function of ship course. At Japan location, Figs. 23, 25 and 27 show the distributions of currents in coils L1–L13, T1–T6, and V1–V6, respectively, while the corresponding data for Chile location are shown in Figs. 24, 26 and 28. The ship's magnetic signature depends on the values of the components of the Earth's magnetic field. The induced magnetization of the ship in three axes is not the same in different parts of the world, which affects the resultant signature.

The optimal coil current includes the DC current and the part changing sinusoidally, as given by the function (8):

8 $$i\left(\varphi \right)={I}_{DC}+{I}_{AMP}sin\left(\varphi +\psi \right)$$

The parameters of the coil currents for the Japan case are presented in Table 4 and for the Chile case in Table 5. It can be seen that changing the ship geographical location forces the change in both the DC part and the amplitudes of the currents.

**Table 4:** Parameters of coil currents for the Japan case.

*coil*	*Ψ * [deg]	*I*_*DC*_ [At]	*I*_*AMP*_ [At]
L1	270	− 532.46	4723.46
L2	270	424.73	1845.64
L3	270	816.38	892.26
L4	270	6.56	708.77
L5	270	− 40.28	883.58
L6	270	− 127.24	633.72
L7	270	251.1	1221.33
L8	90	− 243.26	358.52
L9	270	− 18.53	1106.95
L10	270	− 277.59	350.8
L11	270	− 99.3	573.34
L12	90	152.51	105.2
L13	270	− 14.13	478.07
T1	0	− 20.98	2739.16
T2	0	16.43	226.94
T3	0	− 4.43	1381.27
T4	0	− 60.41	478.79
T5	0	− 8.74	552.25
T6	0	− 14.97	301.26
T7	0	− 64.96	416.33
V1	90	− 408.56	1877.47
V2	270	− 457.16	486.91
V3	90	− 273.16	246.78
V4	90	− 102.3	13.9
V5	270	− 131.14	19.99
V6	90	− 109.29	2.66

**Table 5:** Parameters of coil currents for the Chile case.

*coil*	*ψ* [deg]	*I*_*DC*_ [At]	*I*_*AMP*_ [At]
L1	270	665.14	3077.13
L2	270	− 105.95	1202.35
L3	270	− 687.94	581.27
L4	270	50.95	461.73
L5	270	81.71	575.61
L6	270	121.02	412.84
L7	270	− 597.27	795.64
L8	90	664.96	233.56
L9	270	− 207.54	721.13
L10	270	51.01	228.53
L11	270	− 38.72	373.51
L12	90	− 58.44	68.53
L13	270	− 54.36	311.44
T1	0	16.06	1784.44
T2	0	− 1.63	147.84
T3	0	4.43	899.84
T4	0	− 58.35	311.91
T5	0	13.59	359.77
T6	0	− 30.91	196.26
T7	0	− 65.65	271.22
V1	90	148.13	1223.09
V2	270	455.49	317.2
V3	90	299.05	160.77
V4	90	7.14	9.06
V5	270	− 50.55	13.02
V6	90	14.39	1.73

## Conclusions

The magnetic signature of a ship consists of the induced part and the permanent part. The induced part can be estimated using the FEM and BEM software. Difficulties arise when trying to determine the permanent part of ship magnetization, as there are no direct ways to do it. The measurement of the ship’s magnetic signature gives the total magnetization, therefore the permanent magnetization can only be obtained by subtracting the induced part estimated by the FEM from the complete signature obtained from the measurement. This is why a multi-dipole model that allows the permanent and induced parts to be determined separately from relevant measurements of magnetic signatures in four magnetic directions is so valuable. This paper describes the case of permanent magnetization artificially inserted using DC coils, which was then reconstructed with high accuracy using the multi-dipole model.

Having separated the information on permanent and induced magnetization, it is possible to predict the magnetic signature at any geographical location and for any ship course. The permanent magnetization does not depend on the geographical location, and the induced magnetization can be scaled based on the information about the inclination and magnetic flux density values at a given location in the world. Without separating the two above magnetization components, this would not be possible.

The ability to predict the ship's signature makes it possible to select the currents in the coils such as to minimize the magnetic footprint of the ship at any geographical location. The paper demonstrates a numerical analysis using first the FEM model of a ship to generate its magnetic field with permanent and induced magnetization under conditions corresponding to those in the North Sea. Then, based on this synthetic data, a multi-dipole model of the ship was built to separate the permanent and induced magnetization parts. The induced magnetization was scaled to two different types of conditions representing a completely different geographical location, i.e., near Chile and Japan. The currents in the coils which led to the minimization of the magnetic signatures under these two test conditions were determined, and these currents (along with external magnetic conditions) were applied to the FEM model to generate the signature minimization effect. As a final part of the study, the analysis of coil currents as a function of ship's course was carried out, which revealed the sinusoidal nature of the coil currents and different values of their DC components depending on the external magnetic field.

## Data Availability

The datasets used and/or analysed during the current study available from the corresponding author on reasonable request.
